# A Holistic Analysis of Alzheimer’s Disease-Associated lncRNA Communities Reveals Enhanced lncRNA-miRNA-RBP Regulatory Triad Formation Within Functionally Segregated Clusters

**DOI:** 10.1007/s12031-024-02244-0

**Published:** 2024-08-15

**Authors:** Somenath Sen, Debashis Mukhopadhyay

**Affiliations:** Biophysics and Structural Genomics Division, Saha Institute of Nuclear Physics, A CI of Homi Bhabha National Institute, Kolkata, 700 064 India

**Keywords:** Alzheimer’s disease, lncRNA, miRNA, RBP, Machine learning, ceRNA

## Abstract

**Abstract:**

Recent studies on the regulatory networks implicated in Alzheimer’s disease (AD) evince long non-coding RNAs (lncRNAs) as crucial regulatory players, albeit a poor understanding of the mechanism. Analyzing differential gene expression in the RNA-seq data from the post-mortem AD brain hippocampus, we categorized a list of AD-dysregulated lncRNA transcripts into functionally similar communities based on their *k-*mer profiles. Using machine-learning-based algorithms, their subcellular localizations were mapped. We further explored the functional relevance of each community through AD-dysregulated miRNA, RNA-binding protein (RBP) interactors, and pathway enrichment analyses. Further investigation of the miRNA–lncRNA and RBP–lncRNA networks from each community revealed the top RBPs, miRNAs, and lncRNAs for each cluster. The experimental validation community yielded ELAVL4 and miR-16-5p as the predominant RBP and miRNA, respectively. Five lncRNAs emerged as the top-ranking candidates from the RBP/miRNA-lncRNA networks. Further analyses of these networks revealed the presence of multiple regulatory triads where the RBP–lncRNA interactions could be augmented by the enhanced miRNA–lncRNA interactions. Our results advance the understanding of the mechanism of lncRNA-mediated AD regulation through their interacting partners and demonstrate how these functionally segregated but overlapping regulatory networks can modulate the disease holistically.

**Graphical Abstract:**

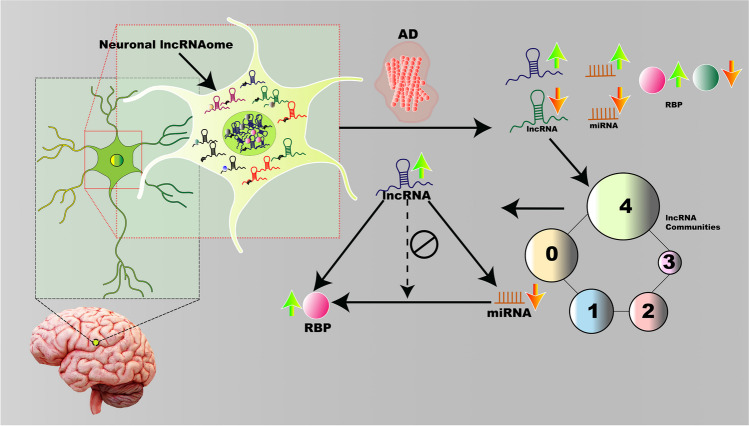

**Supplementary Information:**

The online version contains supplementary material available at 10.1007/s12031-024-02244-0.

## Introduction

Neurodegeneration collectively indicates the chronic progressive aberration of the neuronal structure-function paradigm, where the diverse disease phenotypes arise from location-specific neuronal atrophies (Ising and Heneka 2018; Gan et al. 2018; Andreone et al. 2020; Ou et al. 2021; Lizama and Chu 2021; Shafiq et al. 2021; Wilson et al. 2023). AD-associated neuronal degenerations primarily affect the hippocampus and cerebral cortex and clinically manifest as dementia and cognitive decline (Moreno-Jiménez et al. 2019; Roe et al. 2021; Planche et al. 2022).

Attempts to understand lncRNA-mediated regulation of neurodegenerative conditions, including those in AD, have been gaining importance for more than a decade now (Faghihi et al. 2008; Massone et al. 2011). Recent studies concerning the regulatory pathways implicated in the intracellular imbalance in AD emphasize the importance of lncRNAs as epigenetic regulators. For instance, the β-secretase (BACE1) enzyme that cleaves the amyloid precursor protein (APP) has a natural antisense transcript, BACE1-AS, which upregulates the expression of the enzyme, acting as a lncRNA (Li et al. 2019). The paternally imprinted lncRNA MEG3 shows downregulation in AD rat models and results in disease-associated spatial learning and memory loss and overall neurocognitive deterioration. MEG3 has also been found to rescue neuronal cells in the hippocampus from apoptosis in neurodegenerative scenarios (Yi et al. 2019). NEAT1 and MALAT1, among the nuclear body-associated lncRNAs, have been shown to modulate dysregulated intracellular pathways in AD. NEAT1 is significantly upregulated in AD and impairs PINK1-dependent autophagy, contributing to cognitive decline, and knocking it down reverses the cognitive deficits in AD mice (Huang et al. 2020). In addition to interacting with proteins, lncRNAs also regulate miRNA functionality through sponging (McHugh et al. 2015; Paraskevopoulou and Hatzigeorgiou 2016). Recently, our group has shown how MALAT1 regulates the activity of EPHA2 through miR-200a/26a/26b, eventually attenuating the amyloid-β (Aβ)-induced neurotoxicity (Chanda et al. 2022). Likewise, XIST, known for heterochromatinizing the X chromosome in female cells, sponges miR-132/124 activity, raises BACE1 expression, and aggravates Aβ accumulation in AD (Wang et al. 2018; Yue et al. 2020).

Studies on decoding AD from a broader perspective of a multifactorial disorder involving multiple disease-associated lncRNAs are increasingly relevant in neurodegeneration research (Cao et al. 2019; Huaying et al. 2021). Network dysregulations driven by several non-coding transcripts converge to promote neurodegenerative conditions like AD. To the best of our knowledge, however, the mechanisms by which lncRNAs concertedly influence the dysregulated intracellular networks remain vastly unknown. Through a holistic bioinformatic pipeline developed in this study, we aim to explore how multiple functionally similar lncRNAs act together in an intricate dynamic network in AD. Consequently, the predictions are experimentally validated. Understanding the molecular mechanisms underlying the degenerative processes holds the potential to significantly impact both AD prognosis and therapy.

## Materials and Methods

### Data Acquisition and Pre-processing

The raw RNA-seq data from hippocampal tissues of post-mortem AD brain samples were retrieved from the Gene Expression Omnibus (GEO) repository (Edgar et al. 2002). The search parameters were strictly confined to the pure in vivo LOAD studies from deceased humans, with biological quintuplets as the minimum threshold to ensure uniformity and statistical robustness. Only GSE184942 and GSE173955 among the RNA-seq datasets fulfilled the inclusion criteria and were considered for data acquisition and further analysis. The differentially regulated miRNA set from the human AD brain was obtained from the small RNA-seq dataset of GSE63501 (Table 1). The samples containing FASTQ reads from the tangle-predominant dementia patients were removed from the GSE63501 dataset. It is important to mention that in the small RNA-seq dataset used in this study, the brain tissue region is not known. While the GEO database was initially searched for small RNA-sequencing experiments from human hippocampal samples of AD patients, the database did not return any results.

**Table 1:** In order to obtain a robust list of AD-dysregulated lncRNAs, RNA-seq datasets were used to further enhance the AD-association prediction set (list of the GEO IDs and corresponding information used in the analysis)

S. No.	GEO ID	Platform title	GEO accession	Type	Age	Sex
1	**GSE184942**	Illumina HiSeq 2000 (*Homo sapiens*) (**GPL11154**)	GSM5600937	Control	No data available	No data available
			GSM5600938	Control		
			GSM5600939	Control		
			GSM5600940	Control		
			GSM5600941	Control		
			GSM5600942	AD		
			GSM5600943	AD		
			GSM5600944	AD		
			GSM5600945	AD		
			GSM5600946	AD		
2	**GSE173955**	Illumina HiSeq 2000 (*Homo sapiens*) (**GPL11154**)	GSM5283449	AD	88	Female
			GSM5283450	AD	95	Female
			GSM5283451	AD	95	Female
			GSM5283452	AD	100	Female
			GSM5283453	AD	99	Male
			GSM5283454	AD	83	Male
			GSM5283455	AD	90	Male
			GSM5283456	AD	84	Female
			GSM5283457	Control	87	Female
			GSM5283458	Control	80	Female
			GSM5283459	Control	84	Female
			GSM5283460	Control	77	Male
			GSM5283461	Control	55	Male
			GSM5283462	Control	72	Female
			GSM5283463	Control	78	Female
			GSM5283464	Control	83	Male
			GSM5283465	Control	80	Male
			GSM5283466	Control	74	Male
3	**GSE63501**	Illumina HiSeq 2500 (*Homo sapiens*) (**GPL16791**)	GSM1551173	Control	No data available	No data available
			GSM1551174	Control		
			GSM1551175	AD		
			GSM1551176	AD		
			GSM1551177	AD		
			GSM1551178	AD		
			GSM1551179	AD		
			GSM1551181	AD		
			GSM1551183	Control		
			GSM1551184	Control		
			GSM1551185	Control		
			GSM1551187	Control		
			GSM1551188	Control		

### RNA-Sequencing Data Analysis

To carry out the RNA-sequencing data analysis, first, the raw SRA files were retrieved using the NCBI’s SRA Toolkit (https://hpc.nih.gov/apps/sratoolkit.html) and converted to fastq format. For GSE184942 and GSE173955, the fastq files were only considered with an average read length > 100. The fastq files were analyzed using FastQC (v.0.12.1) (https://www.bioinformatics.babraham.ac.uk/projects/fastqc/) and MultiQC (version 1.15) (Ewels et al. 2016) tools to detect low-quality reads and adapter contaminations. Any residual adapter contamination detected was removed using cutadapt (v.4.4) (https://cutadapt.readthedocs.io/en/stable/). The strandedness of the RNA-seq reads was determined using the RSeQC (version 5.0.1) package (Wang et al. 2012). The reads were then aligned against the GRCh38.p14 using Hisat2 (https://daehwankimlab.github.io/hisat2/). For the GSE63501 dataset, the quality control, read alignment annotation, and analysis were carried out using a small RNA-seq analysis-specific pipeline as outlined in COMPSRA (version 1.0.3) (Li et al. 2020a). In this case, following the default command line, the STAR alignment tool (Dobin et al. 2013) was used to align the reads against the genome index. The count matrix files were generated using featureCounts (version 2.0.1) (Liao et al. 2014). The count data was then analyzed using the DESeq2 package in R to generate the differential gene expression data (Love et al. 2014; Gao et al. 2022). The standard analysis pipeline, as outlined in the original R documentation, was followed.

### Data Post-processing and Sequence Information Retrieval

**Fig. 1:**
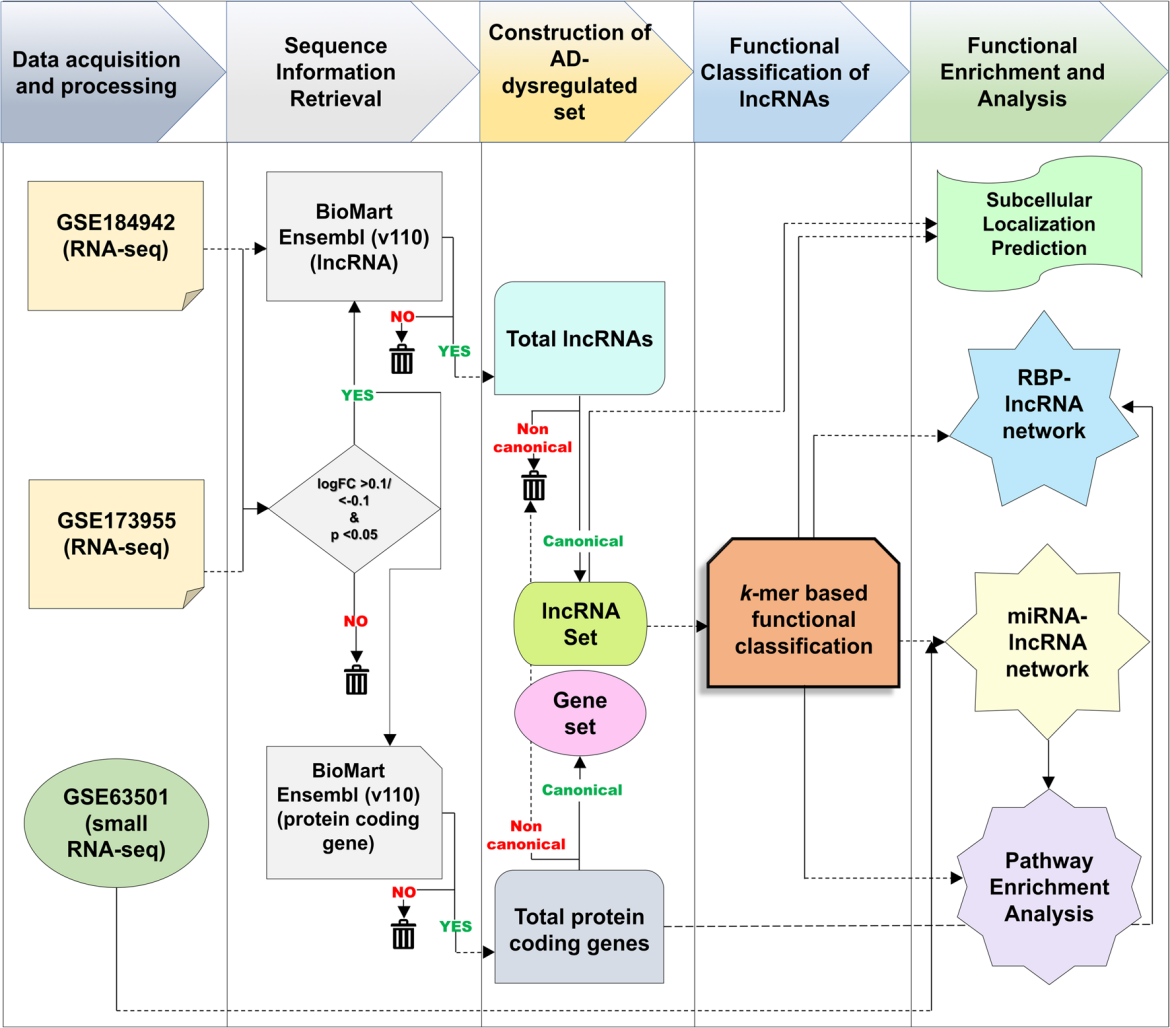
In order to functionally analyze the lncRNAs in the context of their influence in AD, a robust pipeline, as depicted here, was followed, which can be segregated into 5 major steps. Initially, an AD-associated/dysregulated lncRNA set was constructed with the help of RNA-seq datasets GSE184942 and GSE173955. The GSE63501 dataset is the small RNA-seq experiment data that was later used to enhance the lncRNAs for their corresponding AD-dysregulated miRNA interactions. The lncRNAs from the source datasets were passed through a series of filtering parameters, and only the recurrent candidates across all the datasets were chosen for the functional analyses. The lncRNA subset was first functionally classified based on their *k*-mer profiles into functionally resembling communities, and each community was then enriched for the RBP and miRNA interactions, followed by pathway enrichment analysis. The probable subcellular localizations of the lncRNA candidates were predicted using machine learning algorithms

The differential gene expression data obtained from the DeSeq2 analysis were annotated using BioMart (https://asia.ensembl.org/info/data/biomart/index.html) in R. The Ensembl release 110 was used for annotation purposes to identify the gene biotypes. For both the RNA-seq outputs, genes having a log_2_ fold change value greater than (>) 0.1 were considered to be upregulated, and genes having fold change values less than (<) − 0.1 were considered to be downregulated (Fig. 1). Dysregulated genes with a *p* value of less than (<) 0.05 were considered to be statistically significant and suitable candidates for further analysis steps (Tan et al. 2023; Liu et al. 2024).

Genes from each dataset were mapped against the lncRNA and protein-coding genes’ repositories of GENCODE v44 using the Ensembl IDs as the query term (Frankish et al. 2021). The retrieved sequences were further filtered using the Ensembl database (version 110) to obtain the sequence information of the canonical lncRNA transcripts.

### AD-Dysregulated lncRNA, Protein-Coding Gene, and miRNA Set Construction

The individual lncRNA candidates were only selected in the final AD-dysregulated set if they showed statistically significant dysregulation (− 0.1 > log_2_ fold change > 0.1 and *p* value < 0.05) in both the differential gene expression data obtained from GSE184942 and GSE173955. In the case of the protein-coding genes as well, only the common ones between the RNA-seq datasets satisfying the log_2_ fold change and *p* value criteria were chosen for further analyses as the AD-dysregulated set. In the case of the miRNAs, among the differentially expressed miRNAs from the GSE63501 dataset, the statistically significant ones (*p* value < 0.05) were chosen if their log_2_ fold change values lay in the range > 0.1 and < − 0.1 for upregulation and downregulation, respectively.

### Subcellular Localization Prediction of the AD-Dysregulated lncRNAs with Machine Learning

To predict the probable subcellular distribution of each lncRNA from the AD-dysregulated set, three machine learning-based algorithms were run, viz. iLoc-LncRNA (version 2.0) (Su et al. 2018), lncLocator (Cao et al. 2018), and Locate-R (Ahmad et al. 2020). Using the nucleotide sequence of each query lncRNA, these web-based tools utilized support vector machine-based approaches to decipher the probability scores against each lncRNA’s potential subcellular distribution. Only the top-ranked subcellular compartment was chosen for further analysis. With the help of Venn diagram analysis, only the candidates showing similar outcomes across the three pipelines were chosen to be of acceptable confidence.

### *k*-mer Based Community Segregation of AD-Associated lncRNAs

To counter the problem of poor linear sequence homology among lncRNAs, similarities among short motifs of nucleotides called *k*-mers have been suggested to indicate functional similarity. To compute the *k*-mer profiles of the AD-associated lncRNA set, an input file containing FASTA-formatted sequences of the concerned transcripts was created (Supplementary File 1), using which the SEEKR module was run in Python. As suggested by the authors, the value of *k* was chosen to be 6. The adjacency matrix obtained using Pearson’s *r* value was then converted to functionally similar lncRNA communities using the Louvain algorithm. The default commands were used as suggested by the authors with minor modifications (Kirk et al. 2018; Frankish et al. 2021). Since the present versions of the database have moved away from the “− 001” nomenclature to indicate canonical transcripts, utilization of that identifier has been avoided. The GENCODE lncRNA repository was used to retrieve the lncRNA sequence information, and the Ensembl database was used to identify the canonical transcripts according to the latest annotation (version 110).

### RBP–lncRNA Interaction Network Construction of AD-Associated lncRNAs

For each community, the lncRNAs were enhanced for the RBP–lncRNA interactions using only the experimentally validated data from the catRAPID omics (version 2.1) database (Armaos et al. 2021). The interactions were categorized as an “AD” (AD-dysregulated) set and a “comprehensive” set. The AD set was constructed with only the AD-dysregulated RBPs from the GSE184942 and GSE173955 datasets having interactions with the lncRNAs as reported by the catRAPID omics database (Armaos et al. 2021). The comprehensive set was constructed with all the possible experimentally validated interactions against the lncRNAs from each community from the catRAPID omics database. Against each community, two networks were constructed for each interaction dataset using the Cytoscape software (version 3.10.1) (Shannon et al. 2003). For constructing the networks, lncRNAs were denoted as the source node and the RBP interactors as the target node. The log_2_ fold change values of lncRNAs were assigned as the source node attribute. The source nodes were color-coded accordingly using the assigned attribute. The network was analyzed using the Cytoscape network analysis tool.

### miRNA–lncRNA Interaction Network Construction of AD-Associated lncRNAs

The miRNA–lncRNA interaction networks were constructed in a similar fashion as those of the RBP–lncRNA networks against each lncRNA community. To generate the AD-dysregulated miRNA–lncRNA interaction set, altered miRNA expression values from the small RNA sequencing experiment of the human AD brain were deployed (GSE63501). Only the subset of miRNAs present in both the AD-dysregulated miRNA and the output set from the targetscan database against each lncRNA candidate, was chosen as the AD-dysregulated miRNA–lncRNA interactions. The comprehensive miRNA–lncRNA networks, on the other hand, were constructed solely from the targets that can generate outputs without excluding the non-“AD”-dysregulated candidates absent in the AD-dysregulated miRNA list. The targetscan-predicted outputs were further enhanced using the CLIP-seq validated interaction data obtained from the starBase v2.0 database (Li et al. 2014). From the miRNA–lncRNA interaction data, the networks were similarly constructed and analyzed using the Cytoscape software.

### Construction and Analysis of miRNA/lncRNA/RBP Regulatory Triads

For each community, the AD networks of RBP–lncRNA and miRNA–lncRNA interactions were further analyzed to trace potential links within these interactions. First, for each RBP from the AD network, the corresponding CLIP-seq validated miRNA regulators were obtained using the starBase v2.0 database. Next, the RBP–lncRNA interacting pairs were traced, where the interacting miRNA is a common target of both the RBP and the lncRNA from the same pair. A custom R code (Supplementary Data 1) was used to construct the sets of triads. This allowed us to generate a set of regulatory triads for each community. The triads were then depicted using the Cytoscape software.

### Computational Analysis of the miRNA/lncRNA/RBP Regulatory Triad

The RBP/lncRNA interaction was further studied using molecular docking. First, the RBP/lncRNA interaction propensity was predicted using the RPISeq tool, which uses support vector machine (SVM) and random forests (RF) (Muppirala et al. 2011). The lncRNA and RBP structures were then predicted using the AlphaFold 3 (Abramson et al. 2024). Molecular docking was then performed using the Protein Data Bank (PDB)-formatted structures using the HDOCK server (Yan et al. 2017, 2020). The miRNA/lncRNA and miRNA/RBP interactions were studied using the IntaRNA (version 2.0) (Mann et al. 2017) with the FASTA-formatted sequences of these molecules.

### Functional Enrichment Analysis of the lncRNA Communities

Functional enrichment of the lncRNA communities was carried out with the help of interacting miRNAs. From each lncRNA community, the miRNA–lncRNA interaction data obtained from the miRNA–lncRNA networks constructed using the previously described methodology was utilized. The interacting miRNA set from each lncRNA community was enriched using gene ontology enrichment analysis in the miRNA Enrichment Analysis and Annotation Tool (miEAA 2.1) (Aparicio-Puerta et al. 2023). The enriched pathways were sorted based on their adjusted *p* values as well as gene ratios and plotted in the form of a dotplot using the ggplot2 (version 3.4.3) (https://ggplot2.tidyverse.org/) package in R.

### Cell Culture and Transfection

SH-SY5Y (human neuroblastoma) cells were purchased from the Cell Repository of the National Centre for Cell Sciences (NCCS), Pune, India. The cells were maintained following the manufacturer’s instructions in DMEM-F12 (Gibco) media supplemented with 10% (v/v) fetal bovine serum (FBS) (Gibco). Cells were kept in a humified incubator (Thermo) at 37°C with 5% CO_2_. All the transfection experiments were carried out using Lipofectamine 2000 (Invitrogen) reagent, with the cells being 70–80% confluent. The transfection experiments were performed following the manufacturer’s instructions, with the volume of Lipofectamine used being double that of the Plasmid DNA content in micrograms. The transfection efficiency was monitored after 48 h of transfection of GFP-conjugated constructs using a fluorescence microscope.

### Plasmid Constructs and Gene-Specific Primers

The pGFP-C1 (Clontech) (referred to as GFP from here onward) and AICD cloned into pGFP-C1 constructs (referred to as AICD from here onward) were previously available in the lab (Raychaudhuri and Mukhopadhyay 2007, 2010; Raychaudhuri et al. 2012; Baksi et al. 2013; Roy et al. 2014). The primers used in this study are listed in Supplementary Table S1.

### AD Cell Model

SH-SY5Y cells were transfected with either GFP or AICD with Lipofectamine 2000 transfection reagent. The lyophilized Aβ_1–42_ (Sigma A980) was dissolved in dimethyl sulfoxide (DMSO). Three hours post-transfection, the GFP-transfected cells were treated with DMSO, and the AICD-transfected cells were treated with Aβ_1–42_ at a concentration of 0.5 μm. The samples were collected 48 h after the treatment. The GFP-transfected and DMSO-treated cells were considered to be the control set against the Aβ_1–42_-treated cells, transfected with AICD. The latter constituted the AD cell model (Majumder et al. 2017, 2021; Chanda et al. 2021b) (Supplementary Fig. S1).

### RNA Isolation, cDNA Conversion, and Quantitative Real-time PCR

Total RNA isolation from the cells was performed using TRIzol reagent (Invitrogen, USA) following the manufacturer’s instructions. The isolated RNA was quantified using the Nanodrop 2000 microvolume spectrophotometer (Thermo Scientific). The isolated RNA was only considered for further steps if the obtained values of the 260/280 and 260/230 ratios were ~ 2.0, indicating acceptable purity of the yield. To perform the first strand cDNA conversion, 2 μg of RNA was used for each reaction. For coding genes, oligo(dT)18 primers (Thermo Scientific) were used, and in the case of lncRNA genes, random hexamers (Thermo Scientific) were the primers of choice at this stage. For miRNAs, stem-loop primers were used for the reverse transcription stage using the pulse PCR method (Kramer 2011). The cDNA conversions were done using 5× reaction buffer (Thermo Scientific), 10 mM dNTP Mix (Thermo Scientific), oligo(dT)18/random hexamers/stem-loops, and reverse transcriptase enzyme (Thermo Scientific). The qRT-PCR experiments were performed in a QuantStudio™ 3 Real-Time PCR System (Applied Biosystems) using the 2X SYBR™ Green PCR Master Mix (Applied Biosystems), with specific primers (Supplementary Table S1) designed to detect the target genes. The fold change values were calculated by the 2^−ΔΔCt^ method. In the case of non-coding genes, primers against 18S rRNA were used as internal controls, whereas GAPDH was used to normalize coding genes.

### Statistical Analysis

Statistical significance and corresponding *p* values were computed with an unpaired Student’s *t* test using the GraphPad Prism software available at (https://www.graphpad.com/). The *p* values obtained from the RNA-seq data analysis were generated by DESeq2. All the statistical analyses were performed after repeating each experiment at least 3 times. The *p* value of ≥ 0.05 was considered to be indicative of statistically insignificant data and was denoted as “ns”. A *p* value < 0.05 was demarcated as a single asterisk (*), < 0.01 as double (**), and < 0.001 as triple (***).

## Results

### RNA-Sequencing Data from Post-mortem Human AD Brain Identifies Key lncRNA Candidates

**Fig. 2:**
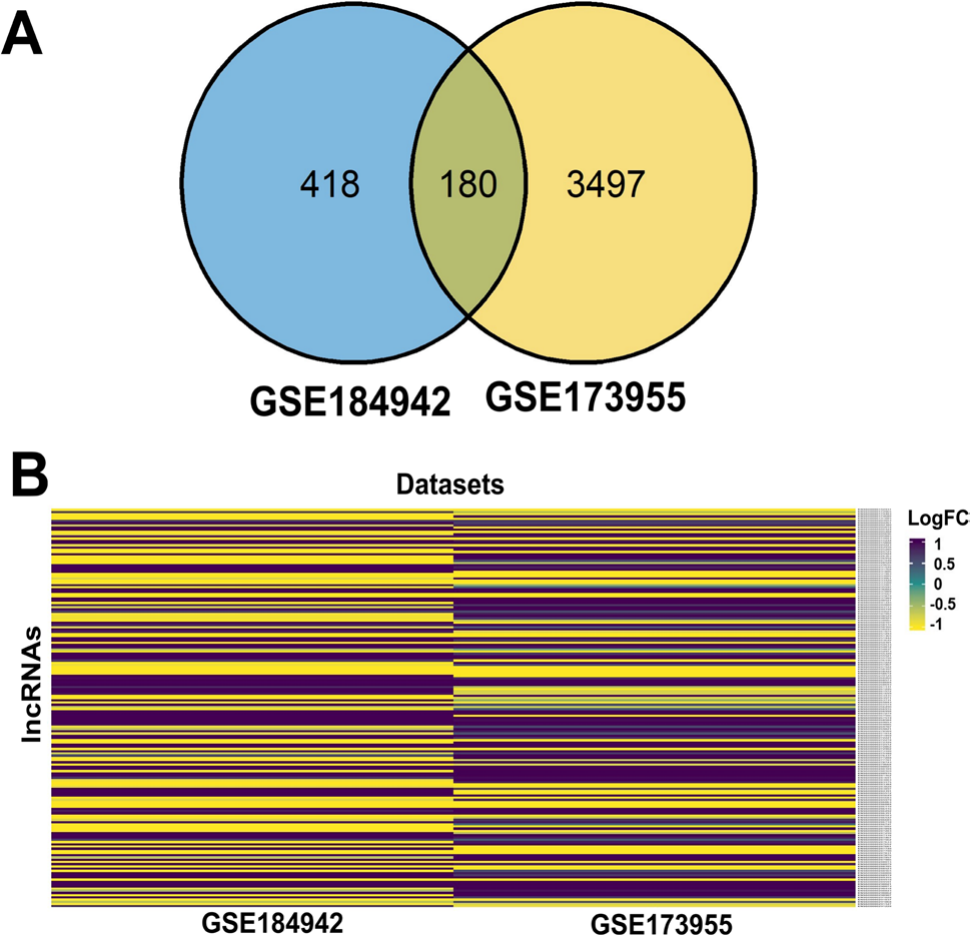
**A**, **B** A Venn diagram analysis from RNA-seq datasets using the Ensembl (version 110) and GENCODE (version 44) as the human lncRNA repository was carried out to delineate the important lncRNA candidates for the functional analysis. The lncRNAs obtained from it contained both canonical and non-canonical variants. For simplicity, from this step onward, only the canonical transcripts were considered for further analysis. The heatmap represents the fold change values of the 180 unique canonical lncRNAs, recurrent across the datasets. It is quite evident from this depiction that among the 180 candidates, quite a few vary in their nature of dysregulation even across these LOAD experiments. This only points towards the significant variation of lncRNA expression levels across the LOAD spectrum. This further complicates the matter of AD diagnosis as well as prognosis in the context of these non-coding transcripts. All of these 180 candidates were statistically significant in both of the RNA-seq datasets

The search criteria, as detailed in the data acquisition part of the “Materials and Methods” section, returned only GSE184942 and GSE173955 datasets. The GSE184942 dataset was composed of 5 controls and 5 AD post-mortem hippocampal tissue samples, although age- and sex-related information were not available. The GSE173955 dataset, on the other hand, was composed of 10 control and 8 AD post-mortem hippocampal tissue samples. While the control cohort had 5 males and 5 females, the AD cohort consisted of 5 females and 3 males. The 28 brain samples (15 controls and 13 AD) in total from the two datasets were used henceforth for performing differential expression analysis. The detailed age and sex distributions of each post-mortem brain sample used have been tabulated further in Table 1. The GSE184942 and GSE173955 datasets returned 43,311 and 37,454 differentially expressed genes, respectively, out of which 2297 and 14,713 genes were statistically significant (Supplementary Tables S2–S5). Using the BioMart annotation scheme and log_2_ fold change criterion (Fig. 1), 598 candidates out of these were found to be lncRNAs from the GSE184942 and 3677 from the GSE173955 datasets (Supplementary Tables S6 and S7). Further analysis yielded 180 lncRNAs as common to both input datasets (Fig. 2A, B.). The lncRNA hits were then scanned against the GENCODE repository (v44) to retrieve the sequence information. Using the Ensembl repository, the lncRNAs were further screened for the canonical transcript variants against each unique lncRNA. Henceforth, only these canonical variants will be analyzed, keeping the simplicity, comparability, and reproducibility of the data in mind. A FASTA-formatted lncRNA sequence set was thus generated, containing the sequence information of the canonical variants of each individual AD-dysregulated lncRNA (Supplementary File 1), and was explored further (Fig. 2A; Supplementary Table S8).

### Sex-Specific Differential Expression Analysis of AD-Associated lncRNAs Reveals lncRNA Subset with Sex-Biased Dysregulation

**Fig. 3:**
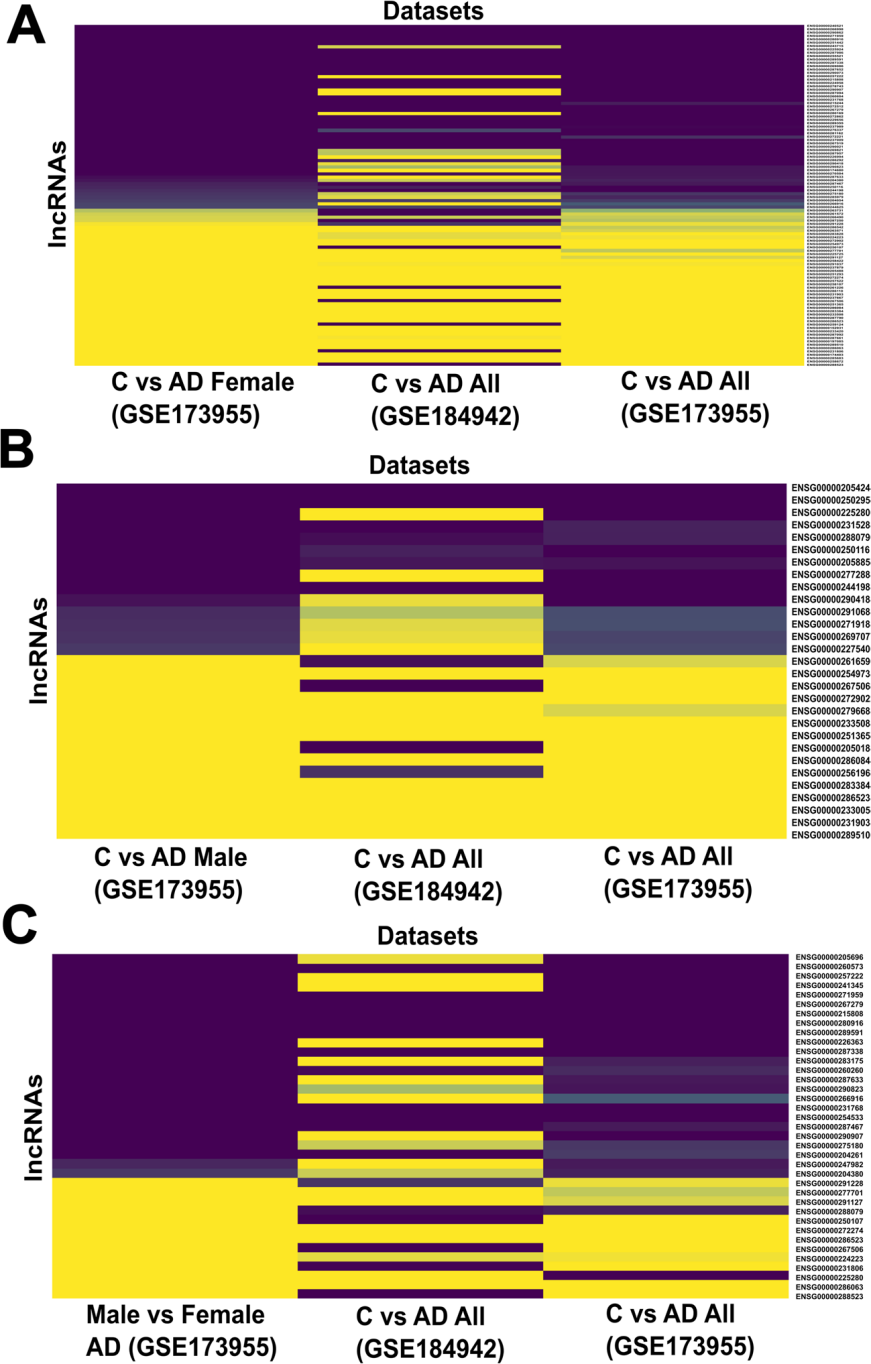
A sex-specific analysis of the 180 AD-associated lncRNAs returned differentially expressed candidates with a sex-biased nature of expression. When the analysis was done among the female samples, 102 lncRNAs were found to be significantly dysregulated (Supplementary Table S9) (**A**). Among the male samples, on the other hand, 29 lncRNAs were found to be differentially expressed in AD (Supplementary Table S10) (**B**). The differential expression analysis was then done to explore the role of gender bias by keeping the phenotype constant. In total, 37 lncRNAs were found to be sex-biased in AD when normalized with control-associated candidates (Supplementary Table S13) (**C**).

The GSE173955 dataset was further used to perform sex-specific differential expression analysis with DeSeq2 with both control and AD cohorts. The sex-biased dysregulation was only considered to be true if the differentially expressed lncRNAs followed the log_2_ fold change and *p *value criteria as outlined in the “Materials and Methods” section, in both control versus AD as well as male versus female scenarios. Initially, the control versus AD differential expression analyses were carried out considering only the male (5 controls and 3 AD) or female (5 controls and 5 AD) samples. Among the female samples, ENSG00000240521 was found to be the most upregulated (4.01 ± 1.79) lncRNA among the 102 female-specific AD-dysregulated candidates (out of the 180 AD-associated lncRNAs), with a similar trend of upregulation found to be present in both the GSE184942 (3 ± 1.52) and GSE173955 (3 ± 0.99) datasets in AD. ENSG00000288523 (− 5.19 ± 1.26), on the other hand, showed the highest downregulation with a similar trend in GSE173955 (− 3.08 ± 0.83), albeit upregulation in the GSE184942 (3.78 ± 1.62) dataset in AD (Fig. 3A; Supplementary Table S9). Among the male samples, 29 lncRNAs out of the 180 were found to be significantly dysregulated in AD, with PCBP3-AS1 emerging as the most upregulated (3.15 ± 1.49) candidate, whereas ENSG00000289510 was found to be the most downregulated (− 4.77 ± 2.02). Both of these lncRNAs showed similar patterns of dysregulation in the GSE184942 (PCBP3-AS: 1.07 ± 0.44; ENSG00000289510: − 4.29 ± 1.7) and GSE173955 (PCBP3-AS: 1.56 ± 0.73; ENSG00000289510: − 2.89 ± 0.77) datasets in AD (Fig. 3B; Supplementary Table S10).

Next, the differential expression analyses were carried out by keeping the disease cohort constant with sex as the variable. The analyses revealed 12 lncRNAs out of the 180 to have sex-biased dysregulation among the control samples (Supplementary Table S11), while the AD cohort revealed 38 lncRNAs to be sex-biased (Supplementary Table S12). The AD cohort revealed ADARB2-AS1 (2.74 ± 0.74) and ENSG00000260573 (2.71 ± 1.36) to be most upregulated in females compared to males. While ADARB2-AS1 showed downregulation in AD in the GSE184942 dataset (− 0.89 ± 0.39), ENSG00000260573 showed significant upregulation in both the GSE184942 (2.68 ± 1.28) and GSE173955 (1.79 ± 0.83) datasets in AD (Supplementary Table S12). Out of the 38 lncRNAs that showed sex-biased dysregulation in AD, except for KCNQ1OT1, no other lncRNA candidates were found to have sex-specific dysregulation in the control cohort. Thus, these remaining 37 lncRNAs out of the 180 can comprise an AD-dysregulated lncRNA subset with sex-specific bias (Fig. 3C; Supplementary Table S13).

### AD-Associated lncRNAs Segregate into Functionally Similar Communities

**Fig. 4:**
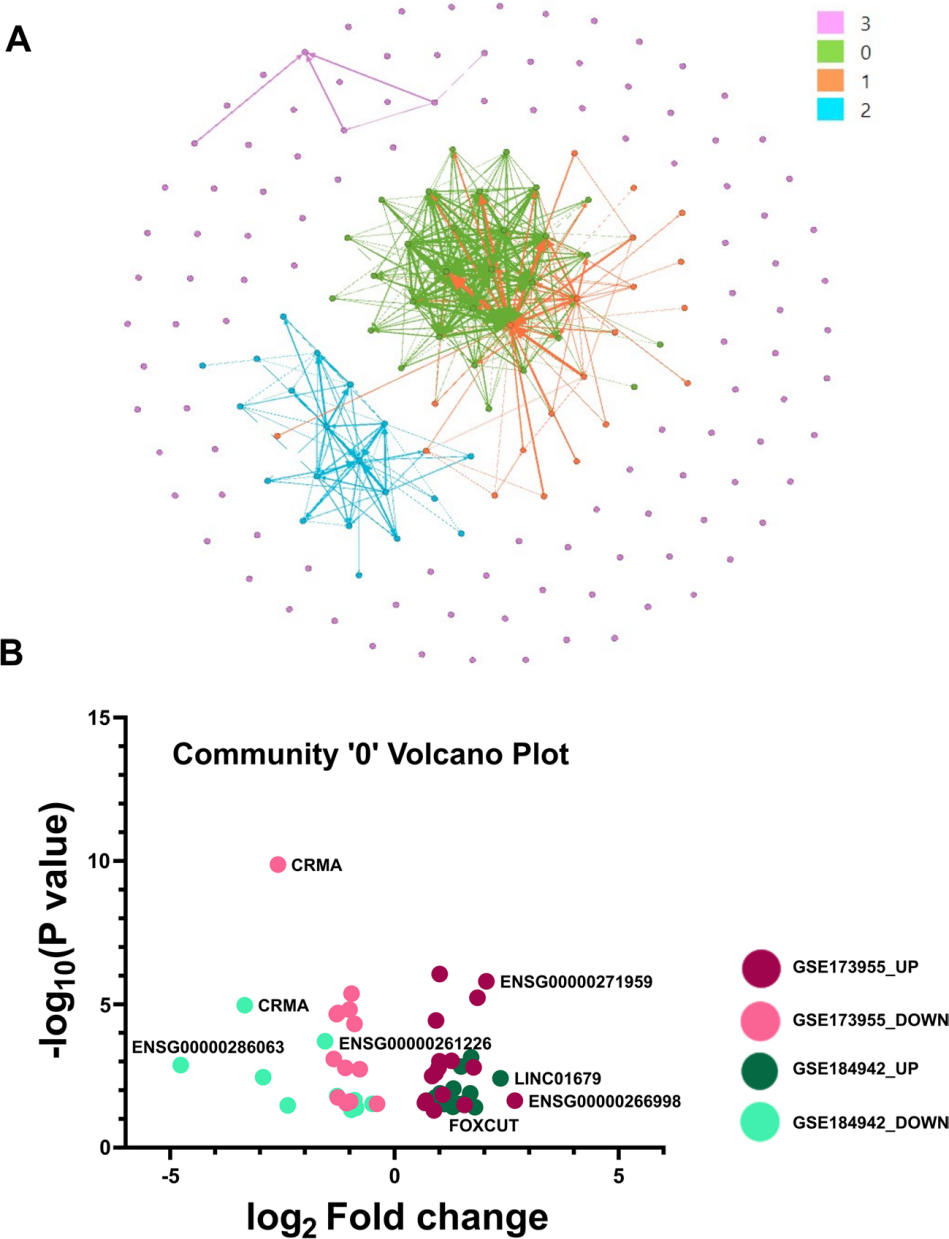
**A** As represented by their corresponding color schemes in the legend, 180 lncRNAs are segregated into 4 communities. Among the four communities, community “0” comprises the highest number of congregated lncRNAs followed by communities “1,” “2,” and “3.” Community 3 consists of the highest number of lncRNAs without any congregation within themselves. **B** Community 0 consists of 29 lncRNAs, which have been depicted in the volcano plot. The red data points indicate the fold change values obtained from the GSE173955 dataset, while the green data points are from the GSE184942 dataset

As compared to proteins, lncRNAs do not share linear sequence homology. Thus, traditional alignment approaches fail to functionally classify lncRNAs. To circumvent this problem, Kirk et al. (2018) suggested that instead of linear sequence homology, lncRNAs having similar *k*-mer profiles share functional similarities. It was further shown that *k*-mer profiles of lncRNAs could be correlated with their localization as well as interacting partners like RBPs. The set of shortlisted 180 lncRNAs (Fig. 2A, B; Supplementary Table S8) were functionally classified based on the *k-*mer profiles of the individual RNAs. Using the SEEKR module, the *k-*mer profiles of each lncRNA (*k* = 6) were computed (Supplementary Table S14). The adjacency matrix obtained using Pearson’s *r* value was then converted to functionally similar lncRNA communities using the Louvain algorithm (Supplementary Table S15). The adjacency matrix was converted into a network to call the communities. In the network, each lncRNA constituted a node, and each “non-zero” element from the adjacency matrix constituted an edge. The unsupervised algorithm yielded communities from the network, wherein the nodes came under a community having more edges among themselves within the community than edges outside it (Supplementary Table S16; Supplementary File 2; Fig. 4A).

The 180 lncRNAs were segregated into 4 communities named “0,” “1,” “2,” and “3.” The 0th community comprised the highest number of functionally clustered lncRNA candidates, followed by the 1st and the 2nd communities. Community 3 consisted of lncRNAs that the algorithm failed to place in any of the clusters (Fig. 4A; Supplementary Table S16) and should therefore be referred to as the “null” community. Although, considering their AD association, their functional relevance should not be too distant from the rest.

It was further observed that, among the 37 lncRNAs with sex-specific bias, lncRNAs present in community 2, contributed 35% of the group, followed by 28% of community 0, 19% of community 1, and 16% of community 3.

### Machine Learning Algorithms Predict that AD-Dysregulated lncRNAs Are Predominantly Cytoplasmic

**Fig. 5:**
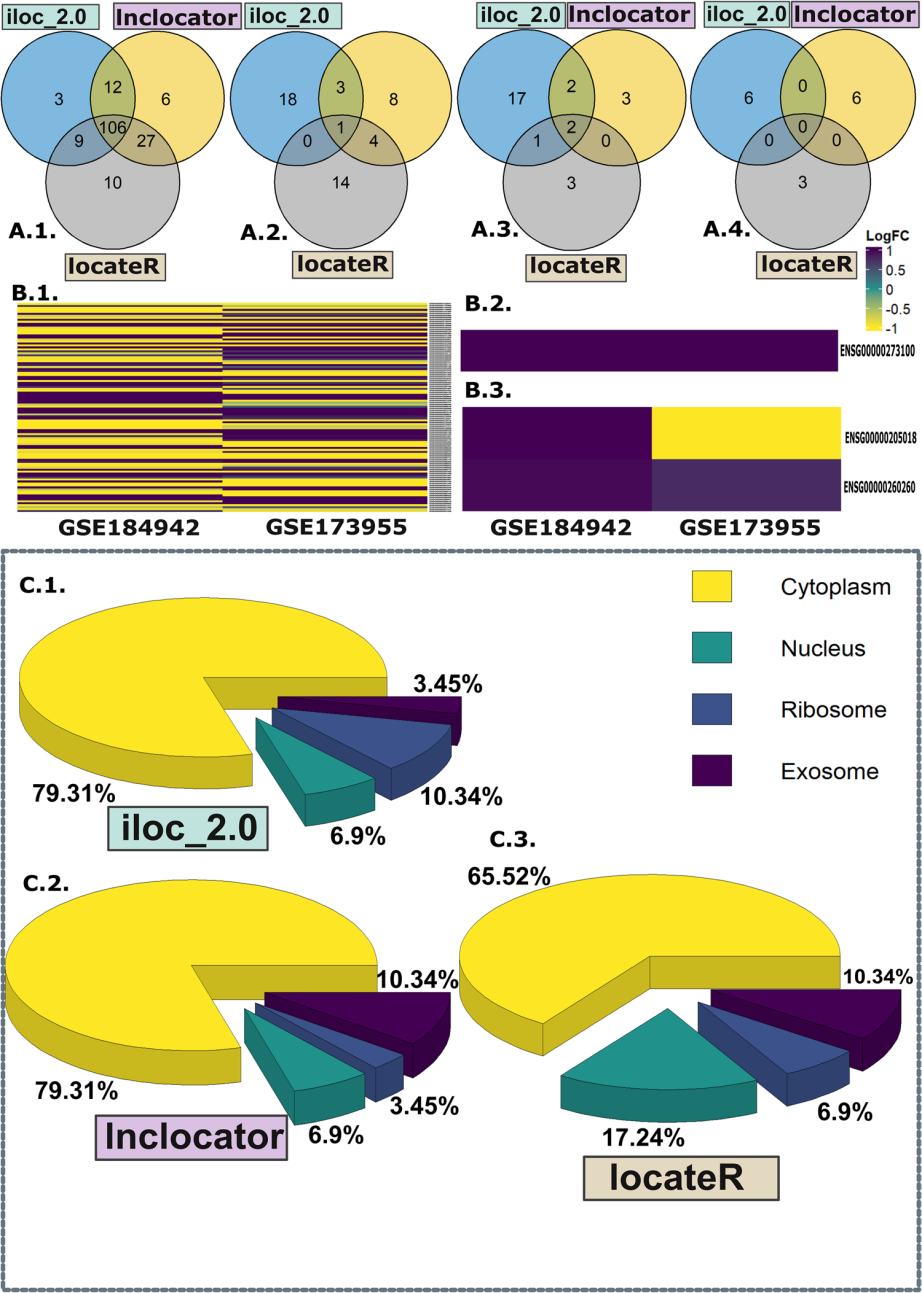
The major driving force behind the rendering of lncRNA-mediated epigenetic regulation of gene expression is their subcellular distribution. To better understand how the AD-dysregulated lncRNAs exhibit their functionality through their subcellular localizations, we used three machine learning-based pipelines to predict their potential subcellular compartment. Among the three prediction outcomes for the subcellular localizations of the lncRNAs from the AD-dysregulated set, even though the cytoplasmic and nuclear RNAs were the top two groups (Supplementary Table S17), there were discrepancies among the three pipelines in assigning the subcellular compartment against each candidate. To address this issue, we performed Venn diagram analyses to find the lncRNAs whose localization propensity was predicted with similar outcomes from all three pipelines. The Venn diagram analyses revealed 106 cytoplasmic lncRNAs (**A** (1)), 1 nuclear lncRNA (**A** (2)), 2 ribosomal lncRNAs (**A** (3)), and 0 exosomal lncRNAs (**A** (4)) to be common. The lncRNA expression profiles for these candidates were also studied, which revealed the nuclear and ribosomal lncRNAs to be significantly upregulated in AD. The cytoplasmic lncRNAs were both up- and downregulated (**B** (2–3)). **C** (1–3) In all three outputs from community 0, cytoplasmic lncRNAs were the most abundant ones, comprising 79% (iloc-lncRNA v2.0), 79% (lncLocator), and 65% (Locate-R) of the candidates. The next most popular subcellular compartment for the community was the exosome with 10% of the total set predicted by both lncLocator and Locate-R. Although Locate-R predicted the nuclear population to be the second highest group, the other two prediction schemes assigned the nuclear lncRNAs a much lower prevalence

The ectopic and/or entopic compartmentalization of lncRNA transcripts allowed these molecules to exert their effects through both upstream and downstream interactions. Machine learning-based subcellular localization prediction methods like iLoc-LncRNA (version 2.0), lncLocator, and Locate-R predicted that 59% of these RNAs have a higher natural propensity towards cytoplasmic distribution based on their nucleotide sequence information (Fig. 5A (1); Supplementary Table S17). We also checked the subcellular localization propensity of each lncRNA from each community, and expectedly, the prediction outcomes were similar, with cytoplasmic lncRNAs emerging to be the leading group (Fig. 5C (1–3); Supplementary Table S17). While the nuclear population was the second highest ranked group in the total set, in community 0, the exosomal population took the second spot in both the lncLocator (10.34%) and Locate-R predictions (10.34%) (Fig. 5C (1–3)). Interestingly, 106 cytoplasmic lncRNAs, 1 nuclear lncRNA, and 2 ribosomal lncRNAs showed similar prediction outcomes across the three algorithms used (Fig. 5A (1–3)). While the common cytoplasmic lncRNAs showed both up- and downregulations (Fig. 5B (1)), the nuclear and ribosomal candidates showed upregulation in the AD brain, with the exception of ENSG00000205018, which showed significantly different results for the two datasets (Fig. 5B (2 and 3)). There were no common exosomal lncRNA candidates with similar outcomes across the three pipelines (Fig. 5A (4)).

### RBP–lncRNA Interaction Networks Help to Identify Important AD-Associated RBP Interactors

RBP–lncRNA interaction networks were constructed considering each individual lncRNA and the experimentally validated RBP–lncRNA interaction data from each community. The same filtered RNA-seq datasets were used to obtain differential mRNA expression information. Following the same scheme as before, the differentially expressed protein-coding genes were retrieved. Out of the 2297 and 14,713 statistically significant genes from the GSE184942 and GSE173955 datasets, 1266 and 9994 were found to be protein-coding, respectively (Supplementary Tables S18 and S19). The common ones were used as a model AD-dysregulated, differentially expressed coding gene set (Supplementary Table S20). The RBPs present in the AD-dysregulated gene set were considered for constructing the AD network, and the comprehensive RBP–lncRNA interaction data were used to compute the comprehensive network. This approach allowed us to generate 2 distinct RBP–lncRNA interaction networks for each of the communities. The comprehensive network comprised both the AD-dysregulated and non-dysregulated RBPs retrieved from catRAPID omics (version 2.1).

**Fig. 6:**
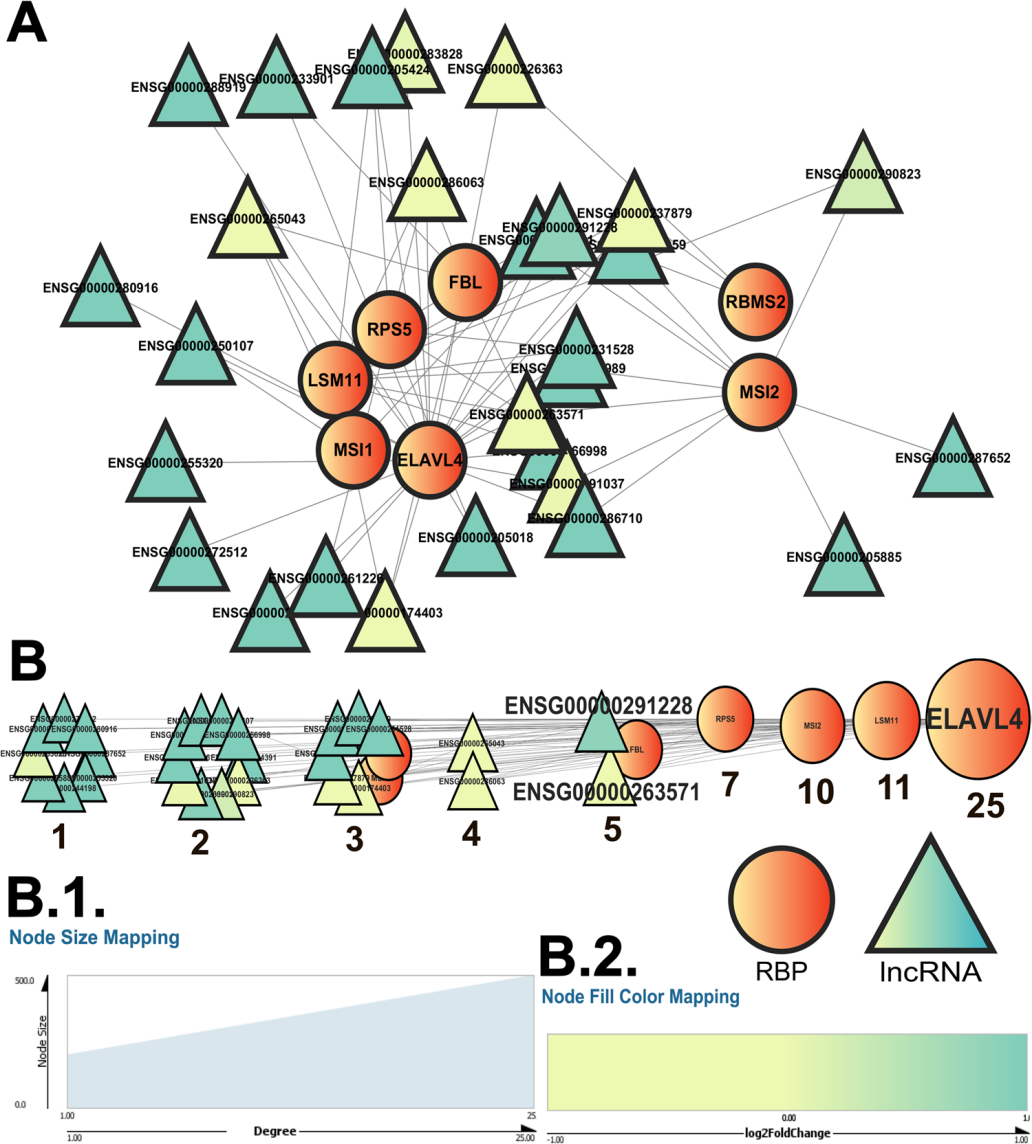
**A** The AD-dysregulated RBP–lncRNA interactions from the catRAPID omics (version 2.1) database (Armaos et al. 2021) gave rise to the RBP–lncRNA “AD” networks. Here, the network emerging from the community “0” (Fig. 4; Supplementary Table S16) has been depicted using the Cytoscape software (Shannon et al. 2003). The network was then further analyzed and sorted based on the degree value of each node (**B**). When multiple nodes share a common degree value, the nodes form a cluster. Each single node and cluster of nodes have been further tagged with their respective degree values, which can be traced to the degree-based rankings of each RBP/lncRNA node as tabulated in Supplementary Tables S21 and S23. In the present network, the top RBP (ELAVL4) and lncRNA (ENSG00000291228 and ENSG00000263571) nodes have been highlighted. These RBP and lncRNA nodes show the highest number of interactions within the nodes of the network. All the nodes of the analyzed network have been portrayed with a size gradient based on their degree values. The scale used to render the respective node sizes has been depicted at (**B** (1)). The log_2_ fold change values for each of the lncRNA nodes from community 0 in the network from the GSE173955 dataset was color-coded following the color gradient scale depicted at (**B** (2)). In both of these networks, the network legends and all the scales have been generated using the Cytoscape software (Shannon et al. 2003). While the ellipsoid nodes represent the RBPs, the triangular nodes represent the lncRNAs

**Fig. 7:**
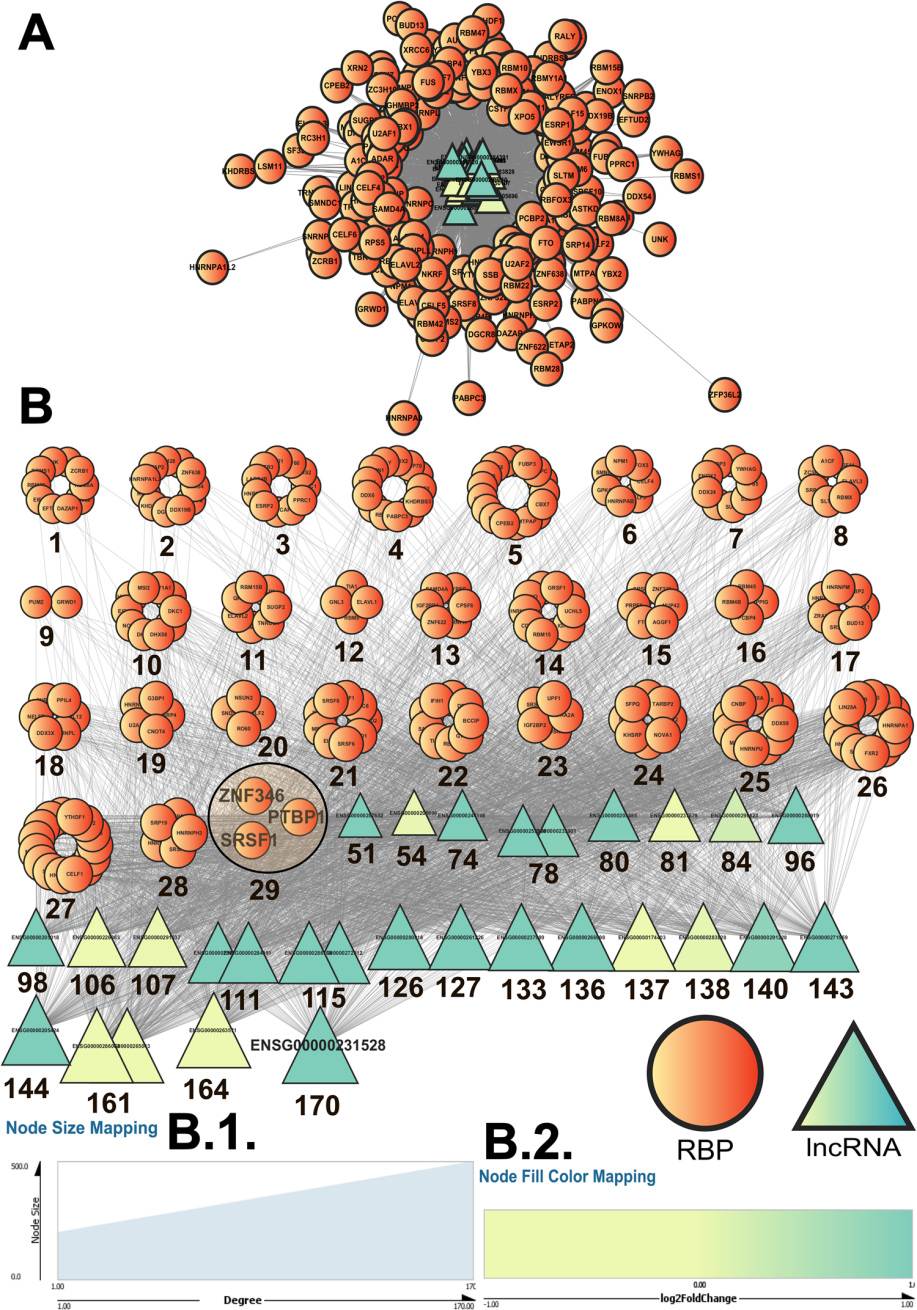
**A** The comprehensive RBP–lncRNA interactions from the catRAPID omics (version 2.1) database (Armaos et al. 2021) gave rise to the RBP–lncRNA “comprehensive” networks. Here, the network emerging from the community “0” (Fig. 4; Supplementary Table S16) has been depicted using the Cytoscape software (Shannon et al. 2003). The network was then further analyzed and sorted based on the degree value of each node (**B**). When multiple nodes share a common degree value, the nodes form a cluster. Each single node and cluster of nodes have been further tagged with their respective degree values, which can be traced to the degree-based rankings of each RBP/lncRNA node as tabulated in Supplementary Tables S22 and S24. In the present network, the top RBP (ZNF346, PTBP1, and SRSF1) and lncRNA (ENSG00000231528) nodes have been highlighted. These RBP and lncRNA nodes show the highest number of interactions within the nodes of the network. All the nodes of the analyzed network have been portrayed with a size gradient based on their degree values. The scale used to render the respective node sizes has been depicted at (**B** (1)). The log_2_ fold change values for each of the lncRNA nodes from community 0 in the network from the GSE173955 dataset were color-coded following the color gradient scale depicted at (**B** (2). In both of these networks, the network legends and all the scales have been generated using the Cytoscape software (Shannon et al. 2003). While the ellipsoid nodes represent the RBPs, the triangular nodes represent the lncRNAs

All these networks were then analyzed using the network analysis tool in Cytoscape. The data allowed us to trace the nodes with the maximum number of edges from each network using degree centrality, and the nodes with the highest degrees were considered to be the most significant. Of those that satisfied the log_2_ fold change and *p* value criterion, 7 (ELAVL4, LSM11, MSI1, MSI2, FBL, RPS5, and RBMS2) had experimentally validated lncRNA interactors from the AD-dysregulated lncRNA set. The AD network of community 0 (Fig. 6; Table 2) had ELAVL4 as the top RBP interacting with 86% of the lncRNAs from within the community and ENSG00000291228 and ENSG00000263571 as the top lncRNAs. The comprehensive network (Fig. 7), on the other hand, had PTBP1, SRSF1, and ZNF346 as the top RBPs, interacting with all the lncRNAs from the community, and ENSG00000231528 as the top lncRNA. The detailed ranking of each RBP and lncRNA on the basis of their degree in the RBP–lncRNA networks for each community is tabulated in Table 2 and Supplementary Tables S21–S24, and the interaction networks for communities 1–3 are depicted in Supplementary Figs. S2–S7.

Despite the variation in the prediction outcomes, quite a few recurring candidates emerged. For AD networks, ELAVL4 appeared to be the most significant RBP as it exhibited the highest number of connections within communities 0, 1, and 3. For comprehensive RBP–lncRNA networks, PTBP1 emerged as the top RBP in communities 0 -3. AD-association being the common factor, this resemblance was expected; however, the degree of these individual RBPs across the communities showed variations in respective rankings (Supplementary Tables S21 and S22).

### miRNA–lncRNA Interaction Network Reveals Potential AD-Associated Interactions

The AD brain small RNA sequencing dataset (GSE63501) allowed us to use the differentially expressed miRNA data to segregate the miRNA–lncRNA interactions from each community into AD and the comprehensive networks. Following the previously explained methodology, the comprehensive network in the case of the miRNA–lncRNA interactions comprised all the potential miRNA-interacting partners for each lncRNA community, including the AD-dysregulated miRNAs. The AD-dysregulated 128 miRNAs obtained from the small RNA-seq dataset (Supplementary Table S25) were used to distinguish the AD-dysregulated target miRNA nodes, giving rise to the AD networks for each lncRNA community. The networks’ analysis then allowed us to trace the top miRNAs and lncRNAs from these interactions using the degree centrality.

**Fig. 8:**
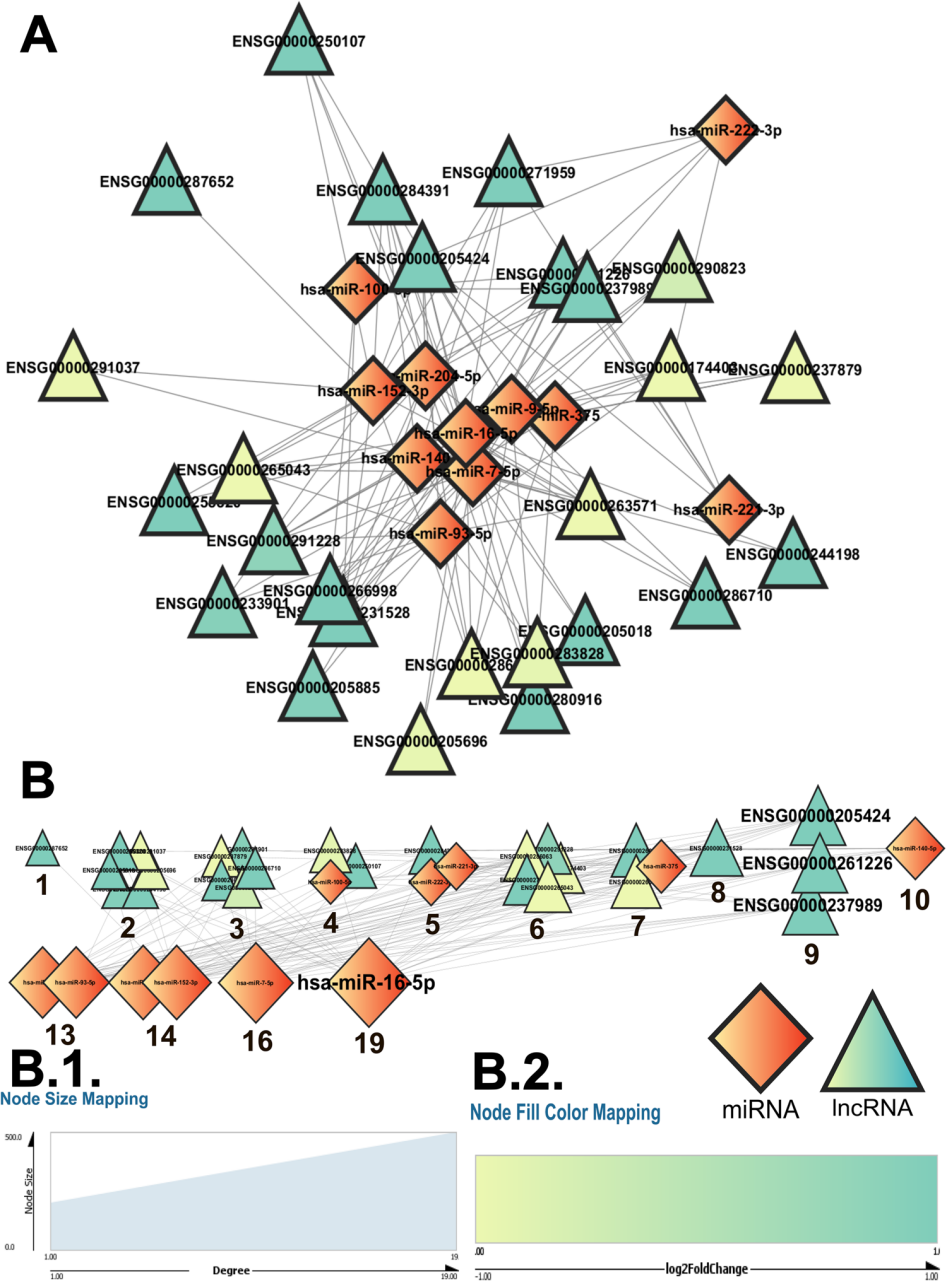
**A** The AD-dysregulated miRNA–lncRNA interactions gave rise to the miRNA–lncRNA “AD” networks. Here, the network emerging from the community “0” (Fig. 4; Supplementary Table S16) has been depicted using the Cytoscape software (Shannon et al. 2003). The network was then further analyzed and sorted based on the degree value of each node (**B**). When multiple nodes share a common degree value, the nodes form a cluster. Each single node and cluster of nodes have been further tagged with their respective degree values, which can be traced to the degree-based rankings of each miRNA/lncRNA node as tabulated in Supplementary Tables S26 and S28. In the present network, the top miRNA (miR-16) and lncRNA (ENSG00000261226, ENSG00000237989, and ENSG00000205424) nodes have been highlighted. These miRNAs and lncRNA nodes show the highest number of interactions within the nodes of the network. All the nodes of the analyzed network have been portrayed with a size gradient based on their degree values. The scale used to render the respective node sizes has been depicted at (**B** (1)). The log_2_ fold change values for each of the lncRNA nodes from community 0 in the network from the GSE173955 dataset were color-coded following the color gradient scale depicted at (**B** (2)). In both of these networks, the network legends and all the scales have been generated using the Cytoscape software (Shannon et al. 2003). While the diamond nodes represent the miRNAs, the triangular nodes represent the lncRNAs

**Fig. 9:**
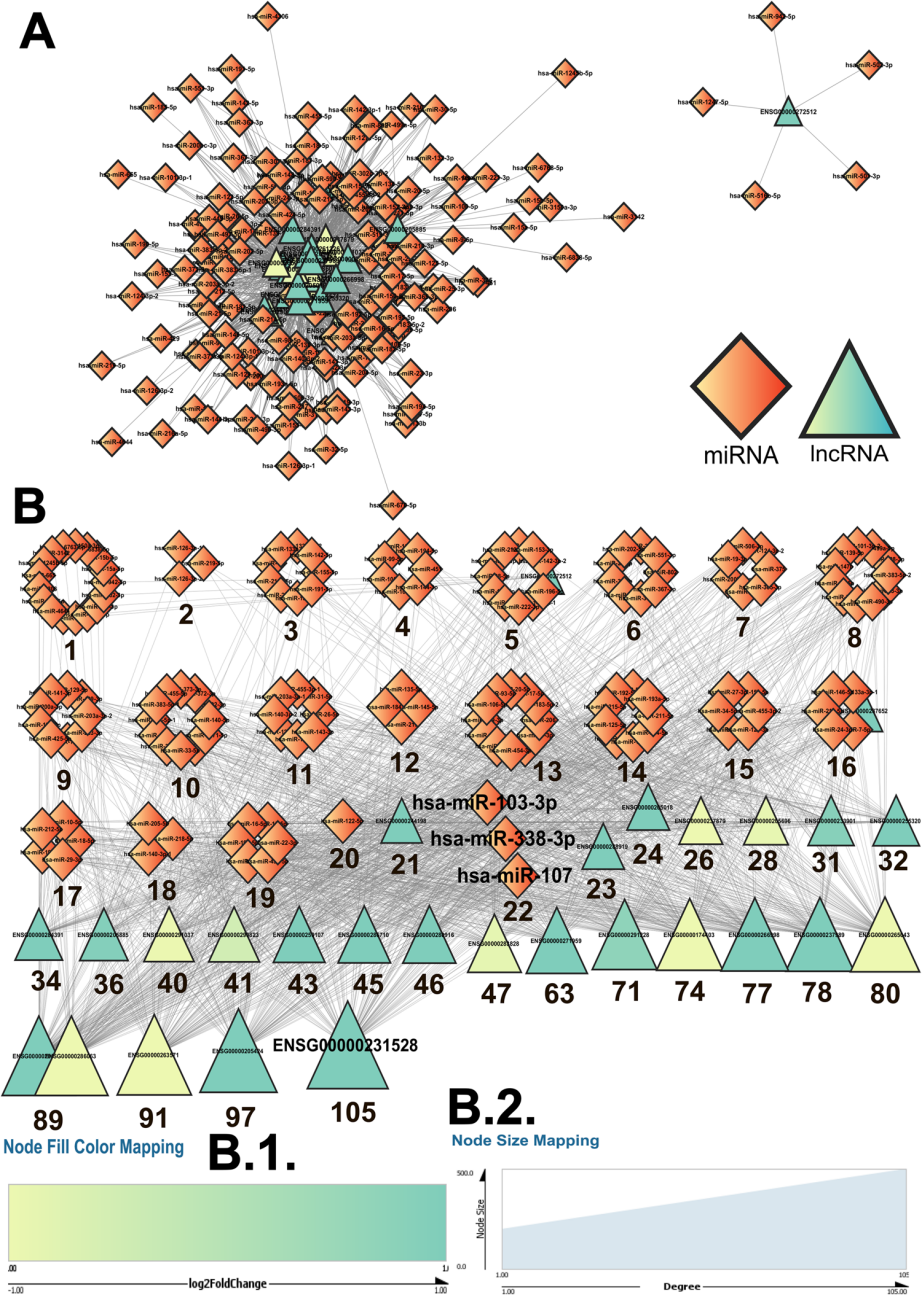
**A** The comprehensive miRNA–lncRNA interactions gave rise to the miRNA–lncRNA “comprehensive” networks. Here, the network emerging from the community “0” (Fig. 4; Supplementary Table S16) has been depicted using the Cytoscape software (Shannon et al. 2003). The network was then further analyzed and sorted based on the degree value of each node (**B**). When multiple nodes share a common degree value, the nodes form a cluster. Each single node and cluster of nodes have been further tagged with their respective degree values, which can be traced to the degree-based rankings of each miRNA/lncRNA node as tabulated in Supplementary Tables S27 and S29. In the present network, the top miRNA (miR-103, miR-338, and miR-107) and lncRNA (ENSG00000231528) nodes have been highlighted. These miRNAs and lncRNA nodes show the highest number of interactions within the nodes of the network. All the nodes of the analyzed network have been portrayed with a size gradient based on their degree values. The log_2_ fold change values for each of the lncRNA nodes from community 0 in the network from the GSE173955 dataset were color-coded following the color gradient scale depicted at (**B** (1)). The scale used to render the respective node sizes have been depicted at (**B** (2)). In both of these networks, the network legends and all the scales have been generated using the Cytoscape software (Shannon et al. 2003). While the diamond nodes represent the miRNAs, the triangular nodes represent the lncRNAs

Community 0, with the highest number of functionally clustered lncRNAs, had miR-16-5p as the top miRNA from the “AD” network (Fig. 8) and miR-103-3p from the “comprehensive” network (Fig. 9) along with miR-107 and miR-338-3p (Table 2). Furthermore, miR-16 ranked second in the AD network and fifth in the comprehensive besides 14 others from community 1. miR-338-3p, the top miRNA from the comprehensive network of community 0, was also at the top in the comprehensive network of communities 2 and 3. The miRNAs 204-5p and 9-5p occupied the top spot in the AD network from community 3 and the third and fourth spots, respectively, in community 0. From the same network, miR-7-5p ranked second, which also happened to occupy the second position in the AD network from community 0 (Table 2; Supplementary Tables S26 and 27). According to the degree of the lncRNAs from the miRNA–lncRNA networks, ENSG00000205424, ENSG00000237989, and ENSG00000261226 were the top lncRNAs from the AD network from community 0. ENSG00000231528 emerged as the top lncRNA from the community 0 comprehensive network (Table 2; Supplementary Tables S28 and 29). The detailed ranking of each miRNA and lncRNA on the basis of their degree in the miRNA–lncRNA networks for each community is tabulated in Table 2 and Supplementary Tables S26–S29, and the interaction networks for communities 1–3 are depicted in Supplementary Figs. S8–S13.

**Table 2:** Each lncRNA community was enriched with RBP and miRNA interactions forming “AD” and “comprehensive” networks. The Top RBPs, miRNAs, and lncRNAs were chosen based on their number of interactions with other nodes within each distinct network. The top nodes are enlisted here for each community with their corresponding degree values. The top lncRNAs originating from the RBP-lncRNA and miRNA-lncRNA networks have been demarcated by ‘a’ and ‘b’ respectively

Communities		RBP	Degree	miRNA	Degree	lncRNA	Degree
Community “0”	AD	ELAVL4	25	hsa-miR-16-5p	19	ENSG00000291228, ENSG00000263571^a^/ENSG00000205424, ENSG00000237989, ENSG00000261226^b^	5^a^/9^b^
	Comprehensive	PTBP1, SRSF1, ZNF346	29	hsa-miR-103-3p, hsa-miR-107, hsa-miR-338-3p	22	**ENSG00000231528**^a, b^	170^a^/105^b^
Community “1”	AD	ELAVL4	17	hsa-miR-9-5p	11	**ENSG00000267519**^a, b^	**7**^a^/11^b^
	Comprehensive	HNRNPH3, PTBP1, SRSF1	21	hsa-miR-24-3p	15	**ENSG00000267519**^a, b^	212^a^/128^b^
Community “2”	AD	LSM11	15	hsa-miR-375, hsa-miR-9-5p, hsa-miR-93-5p	11	**ENSG00000197085**^a, b^	6^a^/10^b^
	Comprehensive	CELF1, DAZ3, ELAVL2, FMR1, FXR1, FXR2, PTBP1, SRP68, SRSF1, ZFP36, ZNF346	20	hsa-miR-203a-3p-2, hsa-miR-338-3p	13	ENSG00000288565^a^/ENSG00000250295^b^	165^a^/97^b^
Community “3”	AD	ELAVL4	46	hsa-miR-204-5p, hsa-miR-9-5p	33	ENSG00000260604, ENSG00000204380, ENSG00000243715, ENSG00000281162^a^/ENSG00000269821^b^	6^a^/29^b^
	Comprehensive	PTBP1	106	hsa-miR-338-3p	43	ENSG00000260604^a^/ENSG00000269821^b^	202^a^/438^b^

### miRNA–RBP–lncRNA Regulatory Triads Are Present in Each Functional Community

**Fig. 10:**
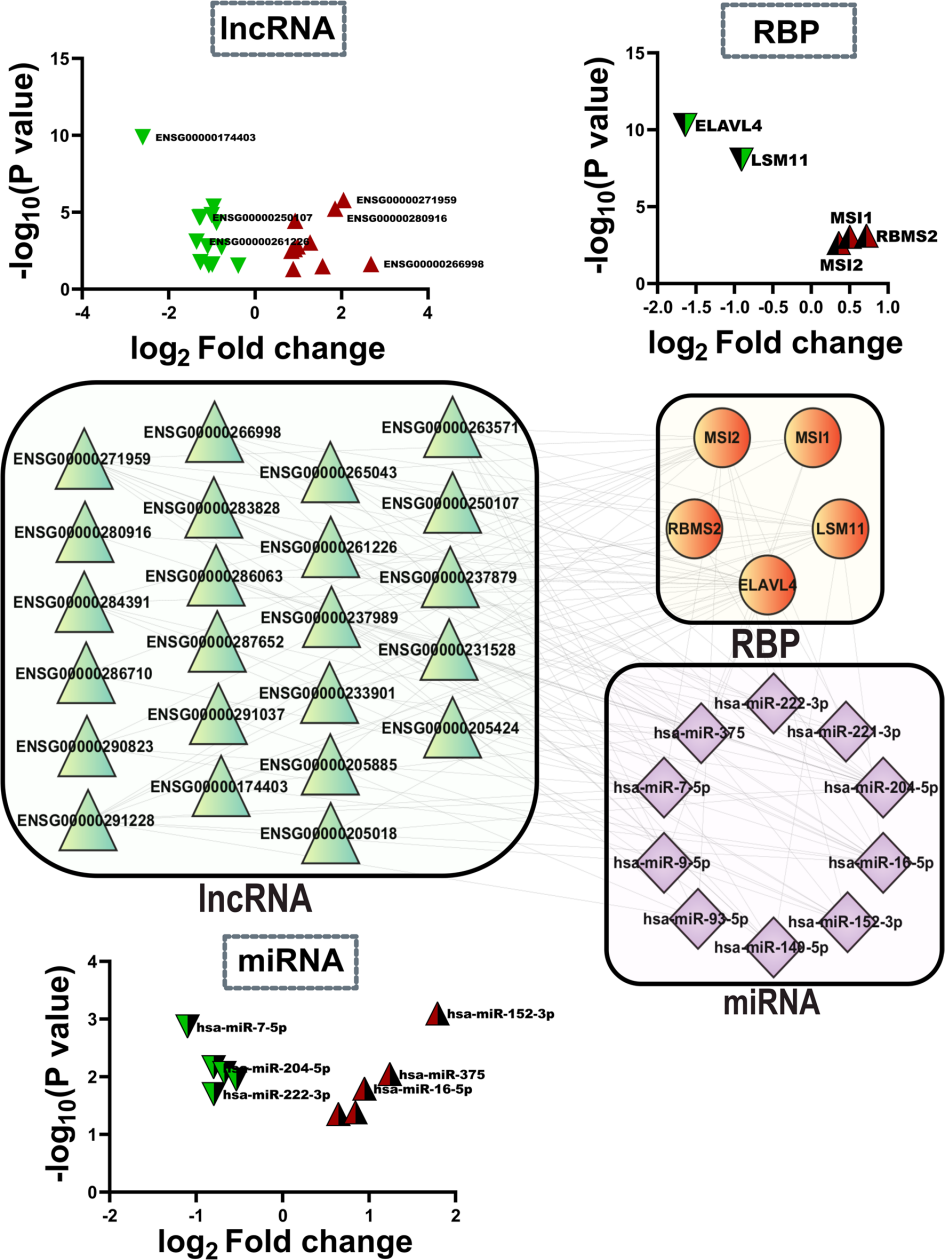
The RBPs from the RBP–lncRNA networks (Fig. 6) were further scanned for CLIP-seq-validated miRNA binding sites. The miRNAs that were found by this approach were then mapped against the miRNA–lncRNA network (Fig. 8) to look for regulatory triads among the miRNA/lncRNA/RBP networks against each community. Here, the regulatory network depicts data originating from community “0”. In total, 79% of the lncRNAs from community 0 participated in the regulatory triads from the same community. One such regulatory triad involves ENSG00000263571, the top lncRNA from the RBP–lncRNA network (Fig. 6; Table 2), which targets miR-16-5p, the top miRNA from the miRNA–lncRNA network (Fig. 8; Table 2). Now, both the lncRNA and the miRNA also have binding sites against LSM11, the second-ranked RBP in the RBP–lncRNA network (Fig. 6). The volcano plots represent the log_2_ fold change values of each lncRNA, miRNA, and RBP from the GSE173955 dataset

The CLIP-seq validated miRNA-RBP interactions obtained from the starBase v2.0 database allowed us to add an additional layer of regulatory information to the RBP–lncRNA and miRNA–lncRNA networks obtained from each AD-dysregulated community. We found 79% of lncRNAs from community 0 interacted with RBPs and miRNAs, provided the miRNAs had reported CLIP-seq-validated interaction sites against the RBP mRNA (Fig. 10; Supplementary Table S30). Among the rest of the communities, community 1 has 57% of its lncRNAs participating in such regulatory triads. Communities 2 and 3 have 65 and 38% of their total lncRNAs, respectively, taking part in forming the said regulatory triads (Supplementary Tables S31–S33).

**Fig. 11:**
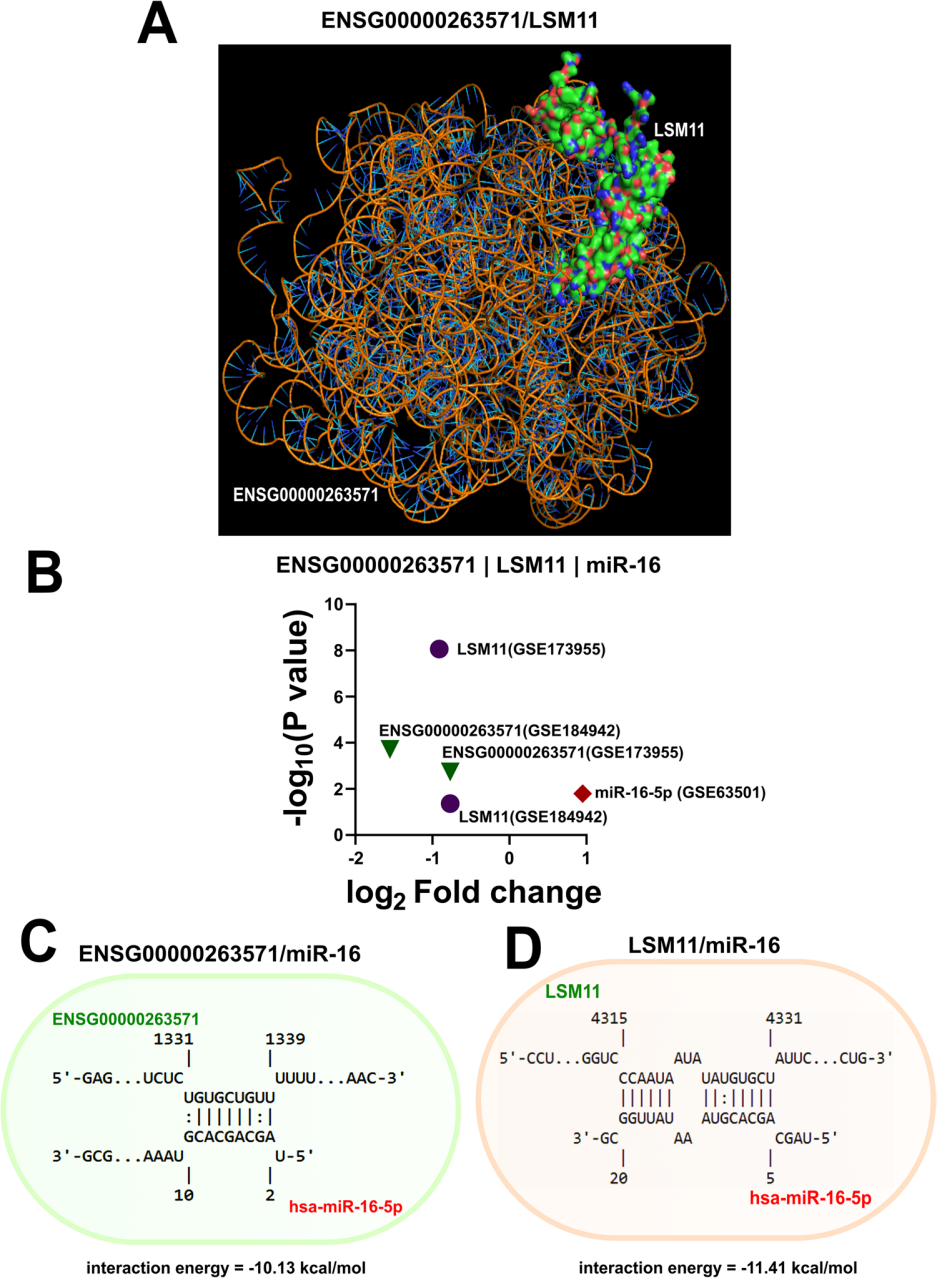
To solidify the possibility of the regulatory triads as predicted previously (Fig. 10.), the interaction propensities among the lncRNA, RBP, and miRNA of one of the regulatory triads (ENSG00000263571/LSM11/miR-16) were further explored. **A** The molecular docking study using the AlphaFold 3 and HDOCK servers returned a high confidence score. The top model of interaction between the molecules has been depicted here. **B** The volcano plot shows the log_2_ fold change values of the lncRNA/RBP/miRNA of this triad. It is assumed that, due to the downregulation of ENSG00000263571 expression in AD, miRNA-16 upregulation is observed, which probably leads to LSM11 downregulation. **C**, **D** To further assess this claim, interaction propensities for miR-16/ENSG00000263571 and miR-16/LSM11 were measured using IntaRNA 2.0. The high negative free energy could allow us to predict the possibility of the said interactions

From these results, possible regulatory triads could be predicted, where functionally similar lncRNA candidates having CLIP-seq-validated binding sites against the AD-dysregulated RBPs also had binding sites against the miRNAs that could otherwise target the said RBP mRNAs. For example, ENSG00000263571 of community 0 formed one such triad with miR-16-5p and LSM11 from the same community (Figs. 10 and 11).

To further solidify this claim, the interaction propensities of these molecules were studied computationally. The RNA-Protein Interaction Prediction (RPISeq) (Muppirala et al. 2011) returned high interaction probabilities between ENSG00000263571 and LSM11. The SVM classifier returned an interaction score of 0.93 in RPISeq. Next, using the AlphaFold 3 model (Abramson et al. 2024), the three-dimensional structures of LSM11 and ENSG00000263571 were generated (Supplementary Files 3 and 4). Using the HDOCK server (Yan et al. 2017, 2020), molecular docking of the structures was performed (Fig. 11A; Supplementary File 5). The top model, with a confidence score of 0.92, had a docking score of − 275.15. The predicted interface residues of the LSM11 and ENSG00000263571 dockings are tabulated in detail in Supplementary Data 2.

The propensities of the miR-16/LSM11 and miR-16/ENSG00000263571 interactions were computed using the IntaRNA 2.0 tool. While miR-16/LSM11 showed an interaction energy of − 11.41 kcal/mol, miR-16/ENSG00000263571 was found to have an interaction energy of − 10.13 kcal/mol (Fig. 11C-D). The high probability of interactions within the miR-16/ENSG00000263571/LSM11 triad adds further confidence in its existence and importance in the context of AD.

### Functional Enrichment of lncRNA Communities Highlights Their Regulatory Roles in AD

**Fig. 12:**
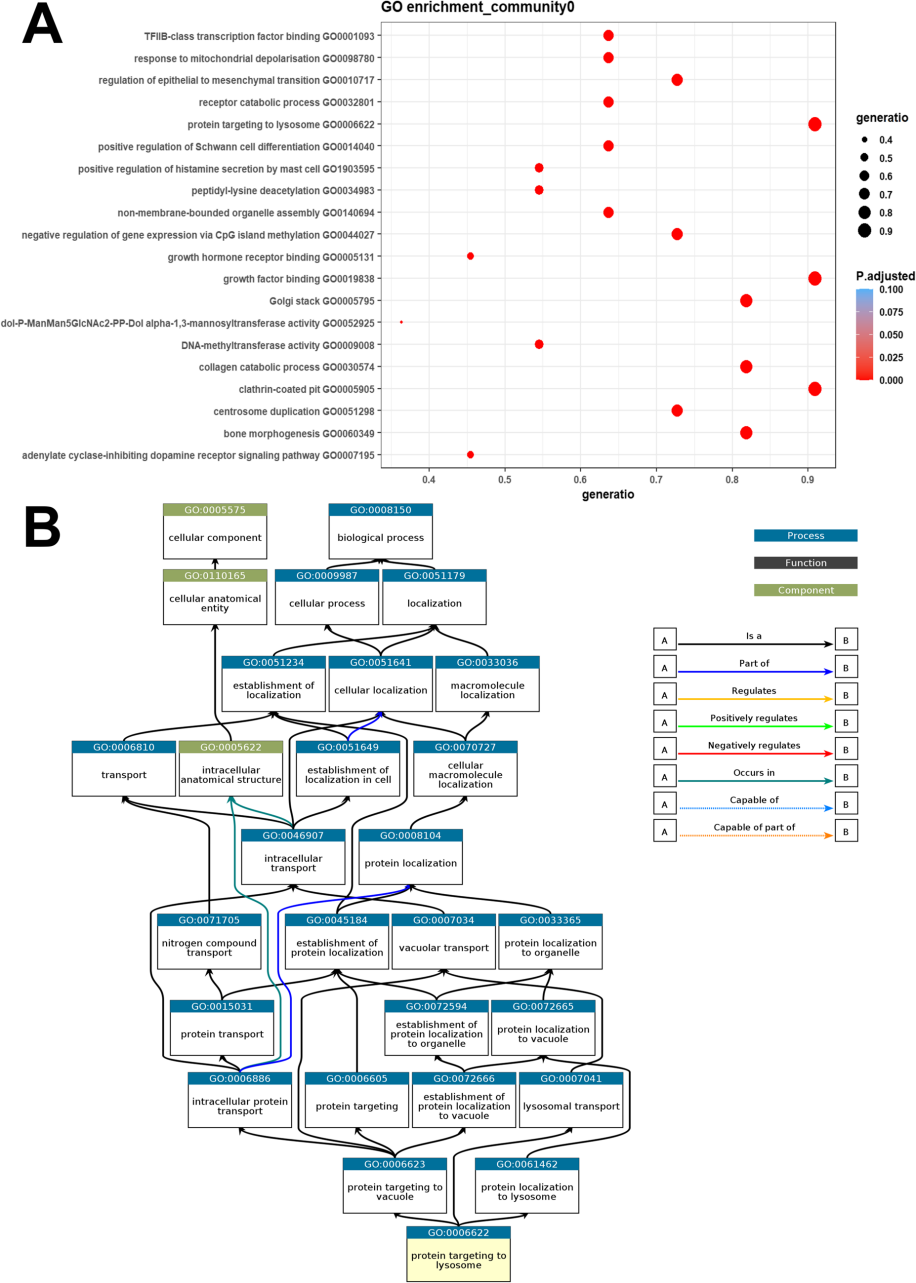
The AD-dysregulated miRNA interactors from community “0” enriched the pathways depicted above in the dotplot. The full list of pathways enriched is enlisted in Supplementary Table S34. All of these top GO terms depicted in the plot were ranked based on their adjusted *p* values as well as gene ratios. While the adjusted *p* values for these top terms were not too far away from each other, the term corresponding to negative regulation of gene expression via CpG island methylation (GO0044027) topped the list (**A**). Whereas, the GO-term protein targeting lysosome (GO0006622) topped the list according to the gene ratio parameter (**B**). This means that the highest number of AD-dysregulated miRNAs from community 0, enriched the said GO term

To understand how the functionally segregated lncRNAs in their respective communities were contributing to the AD phenotype, we performed pathway enrichment from each distinct lncRNA community. The interacting AD-dysregulated miRNAs in each community were thus enriched using miEAA (version 2.1). The enriched GO terms from community 0 included negative regulation of gene expression via CpG island methylation (GO0044027), TFIIB-class transcription factor binding (GO0001093), mitophagy (GO0000423), hippocampus development (GO0021766), insulin-like growth factor receptor signaling pathway (GO0048009), neuron projection extension (GO1990138), etc., to name a few (Fig. 12A; Supplementary Table S34). From this community, the pathway with the highest gene ratio and lowest adjusted *p* value was protein targeting to the lysosome (GO0006622) (Figs. 12B). Another pathway from this community with the highest gene ratio and one of the lowest adjusted *p* values was the regulation of neurogenesis (GO0050767). The top-ranked miRNA miR-16 from community 0 was found to be involved in enriching 79% of the statistically significant pathways from community 0. (Fig. 7; Supplementary Table S34). The topmost enriched terms with the highest gene ratio and lowest adjusted *p* values from the rest of the lncRNA communities were the positive regulation of peptidyl-serine phosphorylation (GO0033138), clathrin-coated pit (GO0005905), and protein stabilization (GO0050821) from communities 1–3, respectively (Supplementary Tables S35–S37; Supplementary Figs. S14–S16).

### Differential Expression of AD-Associated Top lncRNAs, RBPs, and miRNA from the Top Community Could Be Experimentally Validated

**Fig. 13:**
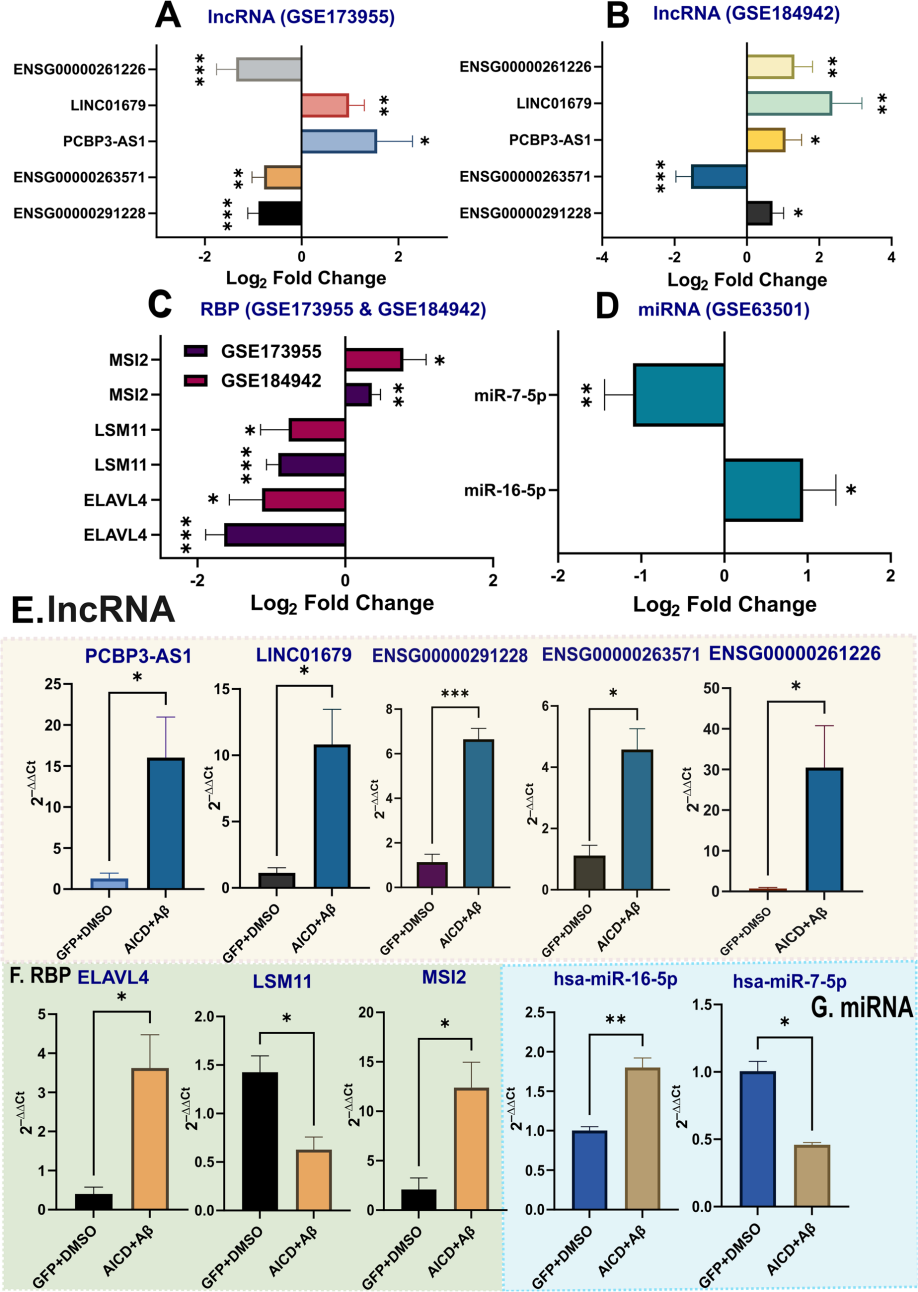
**A**–**C** Detailed analysis of the RBP–lncRNA and miRNA–lncRNA “AD” networks from community “0” establishes PCBP3-AS1 (ENSG00000205424), LINC01679 (ENSG00000237989), ENSG00000291228, ENSG00000263571, and ENSG00000261226 as the top lncRNAs, ELAVL4, LSM11,and MSI2 as the top RBPs, and miR-16-5p and miR-7 as the top miRNAs. Among these top candidates, ENSG00000263571, PCBP3-AS1, LINC01679, ELAVL4, LSM11, and MSI2 showed recurrent themes of dysregulation between the GSE173955 and GSE184942 datasets. The miRNA miR-16 was upregulated while miR-7 was downregulated in GSE63501. **D** All of the top lncRNAs showed statistically significant upregulation in the AD cell model. **E** ELAVL4, the top RBP shows upregulation in the AD cell model; although in both of the RNA-seq datasets, the RBP was significantly downregulated. LSM11 and MSI2, on the other hand, followed the same trend of dysregulation in the AD model as found in the human AD brain. **F** The top miRNA miR-16-5p showed significant upregulation in the AD model, while miR-7-5p was significantly downregulated. The error bars were calculated as mean (fold change) ± SEM value. The *p* value of ≥ 0.05 was considered to be indicative of statistically insignificant data and was denoted as “ns”. *p value < 0.05; **p value < 0.01; and ***p value < 0.001 as triple

Using our in vitro AD model, we validated the dysregulation observed in the top lncRNA, miRNA, and RBPs from community 0 with qRT-PCR. Among the top 5 lncRNAs, only PCBP3-AS1 (ENSG00000205424) and LINC01679 (ENSG00000237989) showed consistent upregulation in both GSE184942 (PCBP3-AS1: 1.07 ± 0.44; LINC01679: 2.36 ± 0.82) and GSE173955 (PCBP3-AS1: 1.56 ± 0.73; LINC01679: 0.98 ± 0.31) datasets, as well as in the AD cell model (PCBP3-AS1: 14.74 ± 4.98; LINC01679: 9.69 ± 2.68). ENSG00000263571 showed downregulation in both the GSE173955 (− 0.77 ± 0.25) and GSE184942 (− 1.55 ± 0.41) datasets; however, the lncRNA was found to be significantly upregulated in the AD cell model (3.46 ± 0.76). Thus, these three lncRNAs appear to have the highest confidence among the top 5 candidates. ENSG00000291228 showed significant upregulation in the GSE184942 dataset (0.71 ± 0.31) and AD cell model (5.51 ± 0.6) and significant downregulation in the GSE173955 dataset (− 0.89 ± 0.22). ENSG00000261226 shows significant upregulation in GSE184942 (1.32 ± 0.5) and in the AD cell model (29.73 ± 10.31) and significant downregulation in the GSE173955 (− 1.35 ± 0.40) dataset. The top RBPs ELAVL4 (GSE184942: − 1.13 ± 0.44; GSE173955: − 1.64 ± 0.25; AD model: 3.22 ± 0.87), LSM11 (GSE173955: − 0.91 ± 0.16; GSE184942: − 0.77 ± 0.38; AD cell model: − 0.7976 ± 0.2128), and MSI2 (GSE173955: 0.36 ± 0.12; GSE184942: 0.79 ± 0.31; AD model: 10.31 ± 2.81) and miRNAs miR-16-5p (GSE63501: 0.95 ± 0.39; AD cell model: 0.79 ± 0.13) and miR-7-5p (GSE63501: − 1.1 ± 0.34; AD cell model: − 0.55 ± 0.07) were experimentally validated as well. While all of the top RBPs and miRNAs showed similar patterns of dysregulation in the AD brain and AD cell model, ELAVL4 showed upregulation in the AD model as opposed to the downregulation observed in the AD brain (Fig. 13).

## Discussion

Understanding the underpinning molecular mechanism in Alzheimer’s disease is still elusive (Hippius and Neundörfer 2003). While it is important to understand how individual molecular players modulate the dysregulated regulatory networks of the disease, a collective understanding of how all these molecules function together, perhaps in dissonance, is of interest.

In recent times, lncRNAs have emerged as key regulatory players that influence various facets of disease processes, to the extent that many of these transcripts are being considered for potential therapeutic targets (Parasramka et al. 2016; Huang et al. 2021; Liu et al. 2022). lncRNAs not only act as sponges, scavenging the miRNA-mediated RNAi pathways, but also act as major architectural molecules that shape the structure–function dynamics of the chromatin and fine-tune the nuclei (Chujo and Hirose 2017). XIST, which has classically established itself as the “X-chromosome inactivating transcript,” is a chromatin-associated lncRNA and alters the chromatin dynamics by recruiting repressive methylation marks like H3K27me3 (Boeren and Gribnau 2021). NEAT1, on the other hand, forms nuclear stress bodies called paraspeckles (Chujo and Hirose 2017; Yamazaki et al. 2018). Each lncRNA, however, is multimodal in its activities downstream, which makes them more interesting. XIST, for example, has been found to be transported to the cytoplasm of female cells upon proinflammatory stress (Shenoda et al. 2021). It is evident therefore that the functional implications brought about by more than a hundred thousand of these transcripts may vary depending on their structural complexity, subcellular localization, and abundance in specific compartments of the cell. It is this structural complexity that is difficult to comprehend owing to the flexible secondary or tertiary structures of these molecules, acting as binding domains for RBPs, small RNAs, mRNAs, and DNAs (Smola et al. 2016; Zampetaki et al. 2018). A multifactorial, complex disease like AD, therefore, has vast implications for how these non-coding transcripts get dysfunctional in their concerted orchestration of cellular homeostasis.

Against this backdrop, we decided to first obtain the AD-associated lncRNA data using publicly available RNA-seq experiments done on post-mortem human hippocampal tissues. Interestingly, quite a few lncRNAs with poor fold change values from the high-throughput analyses have been found to be quite important in AD-like scenarios. For example, MEG3, which shows a fold change of − 0.6 (± 0.21) in the GSE173955 (Supplementary Table S7) dataset, has been reported to significantly improve AD-associated cognitive decline in AD mice models (Yi et al. 2019). MALAT1 shows an even poorer log_2_ fold change value of 0.43 (± 0.16) (Supplementary Table S7) and yet has been found to be important in the context of AD by protecting against Aβ mediated neurotoxicity (Chanda et al. 2022). Moreover, both of these lncRNAs have been found to show opposite modes of dysregulation in AD. While Yi et al. (2019) reported MEG3 downregulation in AD, which was also replicated in our RNA-seq data analysis of the GSE173955 dataset (Supplementary Table S7), other studies (Chanda et al. 2021b; Balusu et al. 2023) found the lncRNA to get upregulated. MALAT1, which was found to be significantly upregulated in our analysis (Supplementary Table S7), has also been previously reported to be downregulated in AD (Ma et al. 2019; Li et al. 2020b). While various factors, including the variety of model systems, could be the reason behind this apparent discrepancy, opposing dysregulation even in human brain samples probably hints toward factors like age, sex, Braak stages, diet, administered drugs and respective dosages, geographical location, genetic variations, etc. While it is beyond the scope of this study to consider all the possible factors at this point, the sex-specific analysis performed on the AD-associated lncRNA set allowed us to gain some insights on sex as a contributing factor for lncRNA-mediated dysregulation in AD (Supplementary Tables S9–S13). Finally, as the present study aims to perform a holistic AD-associated lncRNA functional analysis, a log_2_ fold change value of ± 0.1 was kept at the lowest acceptable cut-off, and the lncRNAs with opposite models of dysregulation in both of the datasets have thus been included as well. It would be prudent to mention that this subset of 180 lncRNAs solely cannot be claimed to be the single most crucial AD-associated/dysregulated set, but a representative set of the significant ones. Further analysis of these lncRNAs should allow us to shed light on their concerted regulatory effect on AD. Furthermore, while adjusted *p* value has been used previously over *p* value for greater stringency (Neff et al. 2021), log_2_ fold change with an unadjusted *p* value of < 0.05 is still widely used by many groups to enable greater inclusion (Crist et al. 2021; Tan et al. 2023; Liu et al. 2024). In the present study, when an adjusted *p* value cut-off of < 0.05 was used as the inclusion criterion along with the log_2_ fold change cut-off of ± 0.1, only 3 lncRNAs (ENSG00000286523, CRMA, and GRIK1-AS1) were found to satisfy both the GSE184942 and GSE173955 datasets simultaneously. This number was too low to perform a meaningful holistic functional analysis, and thus the cut-off of unadjusted *p* value < 0.05 was kept.

It has already been established that lncRNAs function in communities of similar *k*-mer profiles where similar structural RNA motifs could target the degenerate RNA-binding sites in the RBPs. This ensures that these short nucleotide motifs, or *k*-mers, help establish the functional resemblance between lncRNAs with poor linear sequence homology (Kirk et al. 2018). With this idea, the lncRNA subset of our interest was functionally segregated into four clusters (Supplementary Table S16) of similar *k*-mer profiles. It is to be noted that this functional resemblance also renders an additional level of commonality manifested through their interactions with similar RBPs and miRNAs besides AD-association. With 29 lncRNAs clustered together, community 0 was the most populated one and was chosen for experimental validation. Hence, when we analyzed the corresponding interaction network of each community, it was found that in the case of the AD set from community 0, ELAVL4 was shared by 86% and hsa-miR-16-5p was shared by 66% of the lncRNAs from the community (Table 2; Supplementary Tables S21 and S26). ELAVL4 has been found to ameliorate AD-associated Aβ aggregation (van der Linden et al. 2022). Heightened Aβ plaque formation, on the other hand, has been found to increase miR-16-5p levels. This miRNA has been shown to promote neuronal apoptosis in AD by targeting BCL-2 (Kim et al. 2020).

The lncRNAs from each functionally segregated community were further studied using machine learning algorithms to understand their probable subcellular localization. Kirk et al. (2018) originally discussed subcellular localizations and RBP interactions as the shared functions manifested by these functionally segregated communities. Our analyses showed that the commonalities of interactions can be further extended to miRNAs as well. This allowed us to further perform GO enrichment analyses based on the miRNA interactions of each community. Although it is to be noted that in the case of our AD-dysregulated lncRNA set, different communities did not show much dissimilarity between their subcellular localization propensities as compared to the top RBP/miRNA interactions, this, along with the detailed experimentally validated and AD-dysregulated interactions from lncRNA communities, allowed us to perform the holistic functional analysis of the model AD-dysregulated lncRNA set.

These RBPs and miRNAs thus form two of the major focal points (“target nodes”) through which various lncRNAs from each community might channel their regulatory information. Subsequently, these target nodes act as downstream mediators relaying the signals (Ma et al. 2020; Briata and Gherzi 2020). Our results unveil these key functional mediators. It can be further assumed that targeting any of these nodal points with high degree values would significantly impact the interactions downstream. While degree centrality simply reveals the number of interactions each node has, it might not be the sole metric to predict the importance of a node within a network. This study, however, focuses solely on the degree centrality metric to understand which RBPs and miRNAs are majorly used by AD-dysregulated lncRNA communities to disseminate their regulatory signals downstream and subsequently validate a few such genes experimentally. Further detailed analyses with multiple other centrality parameters together could help us further establish the importance of a single node to the flow of information throughout the whole network (Golbeck 2013; Hua et al. 2019).

Among the top AD-dysregulated RBPs and miRNAs, quite a few have already been shown to be implicated in various pathways of AD. Apart from the previously mentioned ELAVL4, Musashi proteins MSI2 and MSI1 have been shown to be present in an oligomeric state in the AD brain with possible colocalization with tau, facilitating neurodegeneration (Sengupta et al. 2018). The top RBPs from the comprehensive network of community 0 PTBP1 and SRSF1 have been found to be linked with suppression of the CD33 isoform associated with a higher risk of AD (van Bergeijk et al. 2019). One of the top miRNAs, miR-7, from communities 0, 1, and 3, has been found to be involved in AD as well. Heightened levels of miR-7 have been shown to result in increased extracellular Aβ; furthermore, the miRNA has been found to inhibit the insulin signaling pathway (Frutos et al. 2019). The top miRNA from community 1, miR-9, has been shown to target the autophagy-associated genes SQSTM1 and OPTN and induce Aβ accumulation (Chen et al. 2021). The top miRNA from the comprehensive network of community 0, miR-107, has been previously shown to accelerate AD progression by its reduced expression as it targets BACE1 (Wang et al. 2008).

It has long been understood that by sequestering the miRNAs, the lncRNAs function as ceRNAs, rescuing the mRNAs from being silenced and degraded (Salmena et al. 2011; Chanda et al. 2021a). Through our study, it has so far been established that the top RBPs and miRNAs are being shared by lncRNAs within their communities; naturally, we wondered whether there lies a further layer of regulatory potential where these top miRNAs are in fact regulating the top RBPs as well. We found that in the case of all the communities generated from the lncRNA set, significant proportions of the community members target miRNAs that, in turn, could potentially regulate their RBP–lncRNA interaction dynamics (Supplementary Table S30–S33). Furthermore, community 0 turned out to be the group with the highest degree of such regulatory triads generated by its lncRNA members. This further strengthens our rationale behind the choice of community 0 as the validation community for the study. Thus, it can be stated that, hrough a regulatory triad, lncRNAs in functionally segregated communities and potentially in various other important functional clusters probably regulate their own binding of RBPs by sponging the miRNAs that the RBPs get targeted by. In this case, based on the RNA-seq data from the GSE173955 and GSE184942 datasets and validation data from the AD-mimicking cell model, it can be hypothesized that, probably by sponging miR-16-5p, ENSG00000263571 could be facilitating its own binding to LSM11 (Fig. 13). According to the experimentally supported miRNA target repository of DIANA-LncBase v3 (Karagkouni et al. 2020), transcriptome-wide miRNA binding site discovery in the human brain carried out by Boudreau et al. (2014) revealed the ENSG00000263571/miR-16-5p interaction. The starBase v2.0 CLIP-seq interaction repository, on the other hand, reported the LSM11/miR-16 interaction from the HITS-CLIP experiment performed by Xue et al. (2013). Finally, the eCLIP interaction reported by the catRAPID omics (version 2.1) database allowed us to predict the ENSG00000263571/LSM11 interaction (Fig. 6). The lncRNA ENSG00000263571 showed significant downregulation in both the GSE184942 and GSE173955 datasets (Supplementary Table S8), while miR-16-5p showed upregulation in GSE63501 (Supplementary Table S25). It could be further assumed that, due to this upregulation, probably the RBP LSM11 showed significant downregulation as well (Supplementary Tables S18 and S19). Further computational analysis with molecular docking to study LSM11/ENSG00000263571 interactions and RNA–RNA interaction studies to compute the interaction propensities of miR-16/ENSG00000263571, miR16/LSM11 were performed (Fig. 11). While these predicted triads are novel at this point, our analyses could be the basis on which further validation of these interactions could be pursued in AD. The exact roles of such triads, apart from the functionally relevant interactions of these AD-associated biomolecules, are yet to be properly understood. The LSM11 has been reported to be involved in pre-mRNA processing (Pillai et al. 2001), which could be hindered by miR-16 interaction. This further solidifies the role of miR-16 as a potentially harmful miRNA in AD. However, the importance of the LSM11/ENSG00000263571 interaction in AD is not clear.

Since lncRNA functionality is guided through its subcellular distribution apart from its intracellular and extracellular interactions, it can be assumed that the regulatory triads are guided by the altered subcellular distribution of the AD-dysregulated lncRNAs from each community. It is beyond the scope of this study to further validate the said interactions through fractionation and pull-down experiments, which could shed more light on the role of these triads in AD.

With 79% of the candidates forming these regulatory triads, lncRNAs from community 0 appear to be more inclined towards targeting miRNAs that would in turn enhance their target AD-dysregulated RBP binding. It is also interesting to note that apart from community 3, all the other communities seem to regulate 5 of the 7 RBPs that satisfy the AD dysregulation cut-off. While FBL was only regulated by community 3, none of the 4 communities showed any tendency toward facilitating RPS5 expression through the proposed mechanism.

## Conclusion

Studying Alzheimer’s disease regulation could be the key to developing novel therapeutic strategies that are currently lacking. With the advent of newer techniques to study and target lncRNAs, that gap could potentially be bridged. The present study allows us to focus on not only the importance but also the extent to which lncRNAs cumulatively influence AD as a whole. While gene editing techniques allow us to inhibit target genes to tackle their deleterious effects, targeting a single gene in the vast, intricate intracellular network is often challenging from the point of view of prognosis and efficacy. Thus, it is imperative to unravel hubs that act as converging points for the dysregulated genes around them to target complicated conditions like AD. Although it is beyond our present capability to target the huge number of lncRNAs in their dynamically dysregulated homeostasis, understanding their varying influence and importance in AD could help us guide toward the direction of efficacious therapeutic interventions.

## Supplementary Information

Supplementary Figure S1 (PNG 966 kb)

High resolution image (TIFF 106347 kb)

Supplementary Figure S2 (PNG 1254 kb)

High resolution image (TIFF 191987 kb)

Supplementary Figure S3 (PNG 4052 kb)

High resolution image (TIFF 238391 kb)

Supplementary Figure S4 (PNG 1193 kb)

High resolution image (TIFF 214269 kb)

Supplementary Figure S5 (PNG 4607 kb)

High resolution image (TIFF 375513 kb)

Supplementary Figure S6 (PNG 1808 kb)

High resolution image (TIFF 197450 kb)

Supplementary Figure S7 (PNG 4469 kb)

High resolution image (TIFF 372376 kb)

Supplementary Figure S8 (PNG 1257 kb)

High resolution image (TIFF 223468 kb)

Supplementary Figure S9 (PNG 4076 kb)

High resolution image (TIFF 222695 kb)

Supplementary Figure S10 (PNG 1220 kb)

High resolution image (TIFF 185360 kb)

Supplementary Figure S11 (PNG 2643 kb)

High resolution image (TIFF 223944 kb)

Supplementary Figure S12 (PNG 2316 kb)

High resolution image (TIFF 225679 kb)

Supplementary Figure S13 (PNG 5486 kb)

High resolution image (TIFF 373106 kb)

Supplementary Figure S14 (PNG 1496 kb)

High resolution image (TIFF 231150 kb)

Supplementary Figure S15 (PNG 852 kb)

High resolution image (TIFF 190084 kb)

Supplementary Figure S16 (PNG 839 kb)

High resolution image (TIFF 192167 kb)

ESM 17 (FA 359 kb)

ESM 18 (GML 52 kb)

ESM 19 (PDB 219 kb)

ESM 20 (PDB 6397 kb)

ESM 21 (PDB 4518 kb)

ESM 22 (DOCX 12 kb)

ESM 23 (DOCX 19 kb)

ESM 24 (XLSX 9 kb)

ESM 25 (XLSX 7870 kb)

ESM 26 (XLSX 7773 kb)

ESM 27 (XLSX 18 kb)

ESM 28 (XLSX 1168 kb)

ESM 29 (XLSX 42 kb)

ESM 30 (XLSX 27 kb)

ESM 31 (XLSX 1223 kb)

## Data Availability

The datasets used in this study are freely available at the NCBI’s Gene Expression Omnibus database (https://www.ncbi.nlm.nih.gov/geo/). The codes used in aiding the analysis steps are available upon request to the authors.
