# Supplementary figures and images for: A Holistic Analysis of Alzheimer’s Disease-Associated lncRNA Communities Reveals Enhanced lncRNA-miRNA-RBP Regulatory Triad Formation Within Functionally Segregated Clusters
Source: J Mol Neurosci. 2024 Aug 15;74(3):77. doi: 10.1007/s12031-024-02244-0 (PMC11324768; doi:10.1007/s12031-024-02244-0)

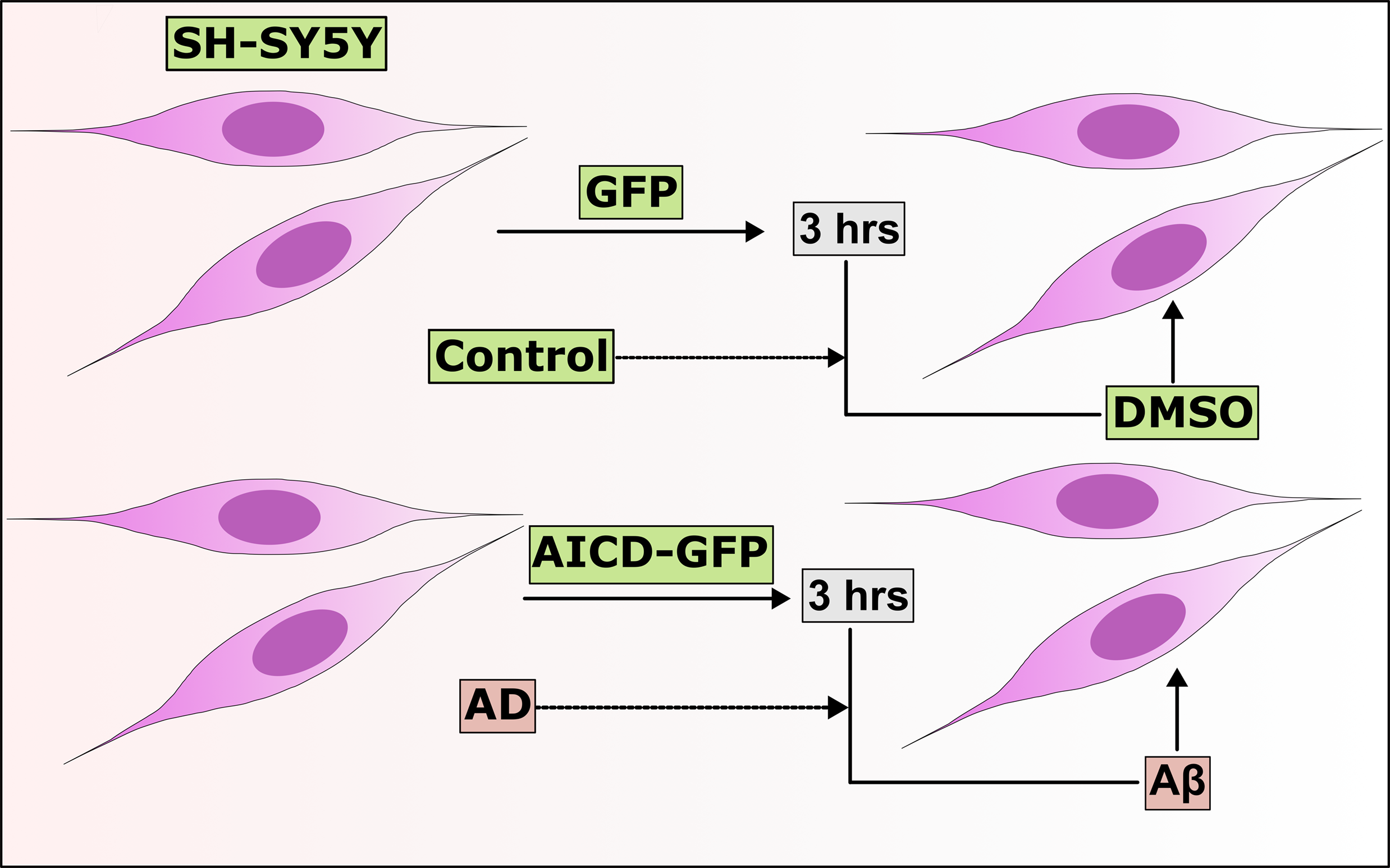

Supplement: Supplementary file 1 — (PNG 966 kb) [file 12031_2024_2244_Fig14_ESM.png]

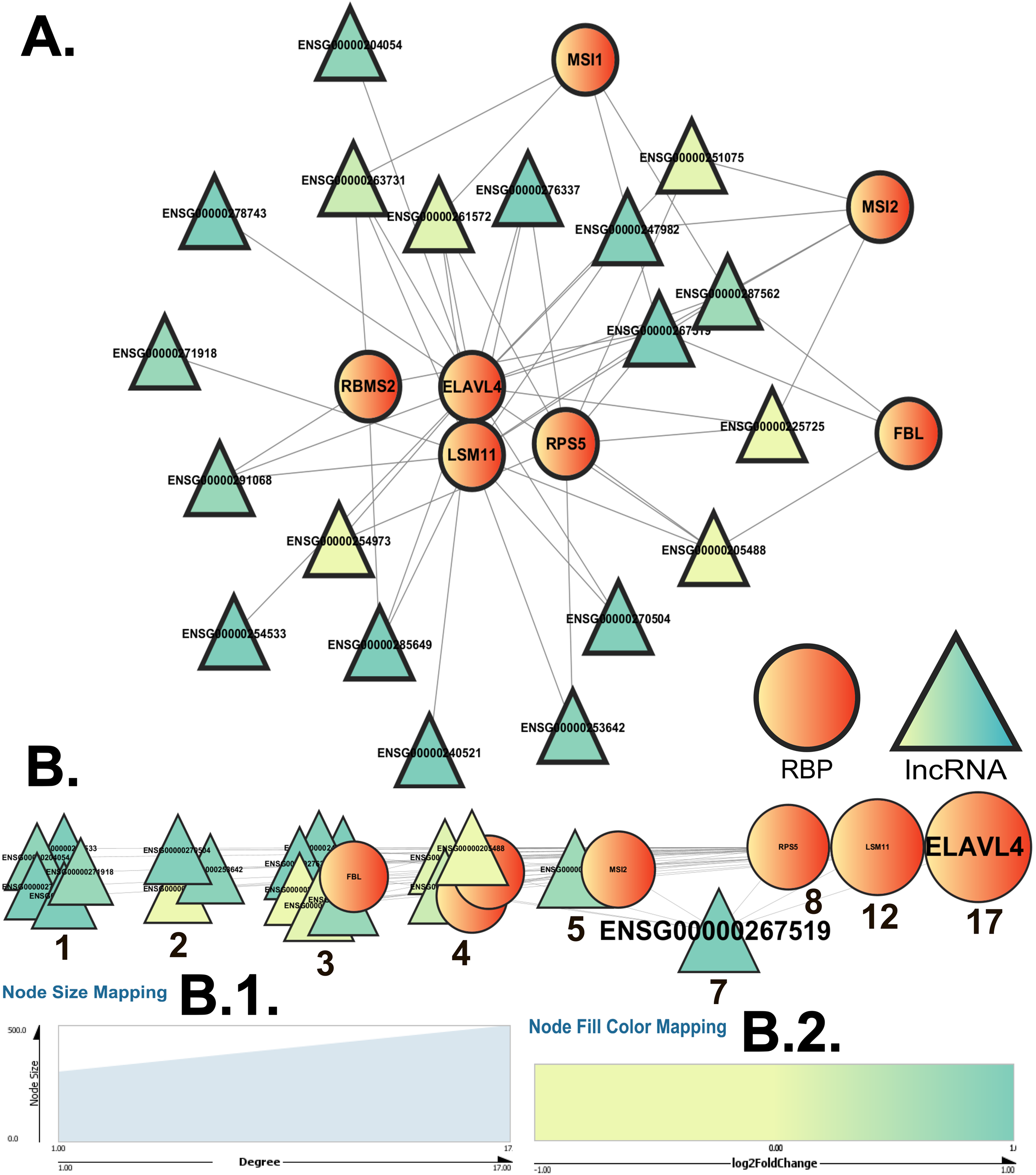

Supplement: Supplementary file 3 — (PNG 1254 kb) [file 12031_2024_2244_Fig15_ESM.png]

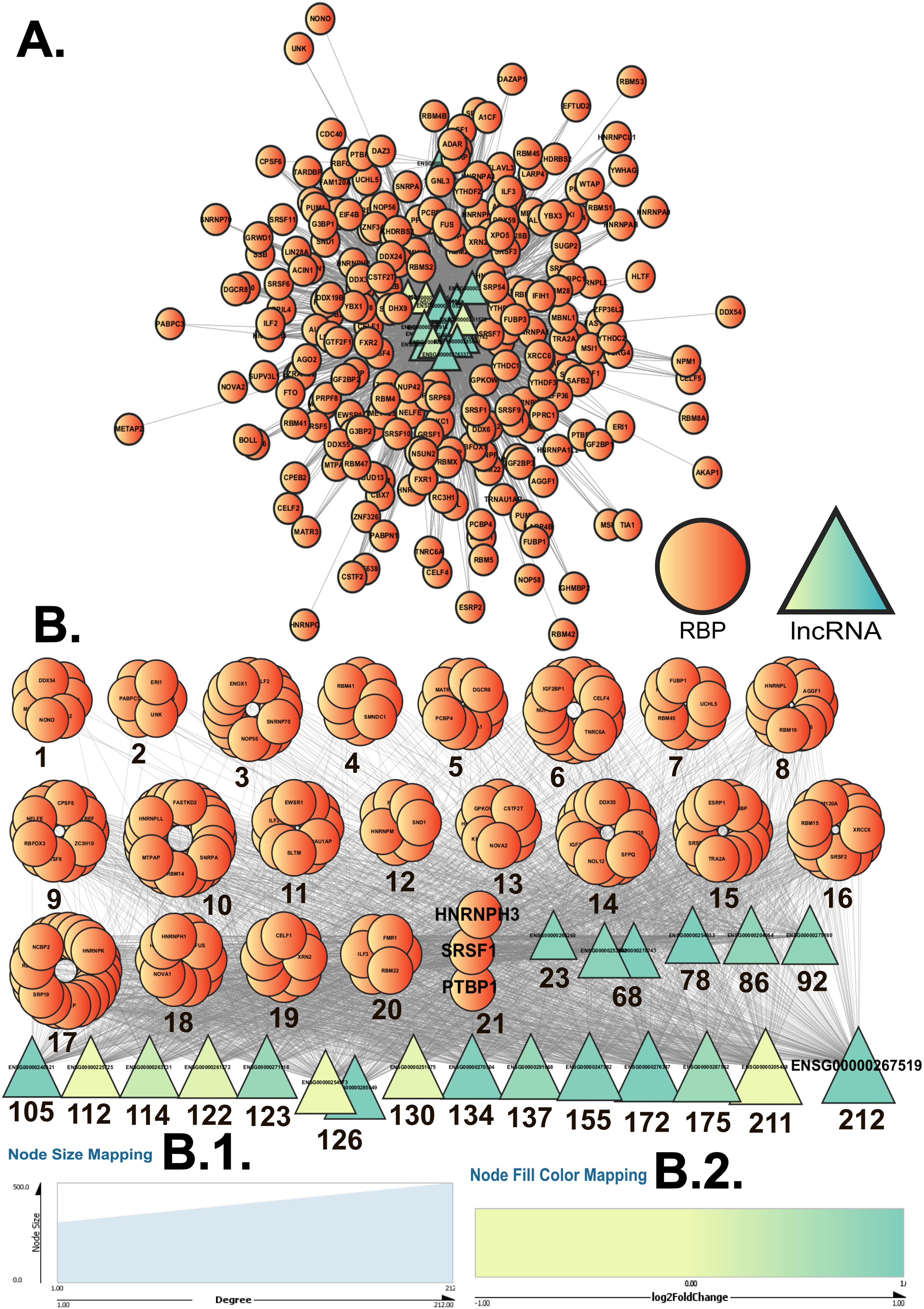

Supplement: Supplementary file 5 — (PNG 4052 kb) [file 12031_2024_2244_Fig16_ESM.png]

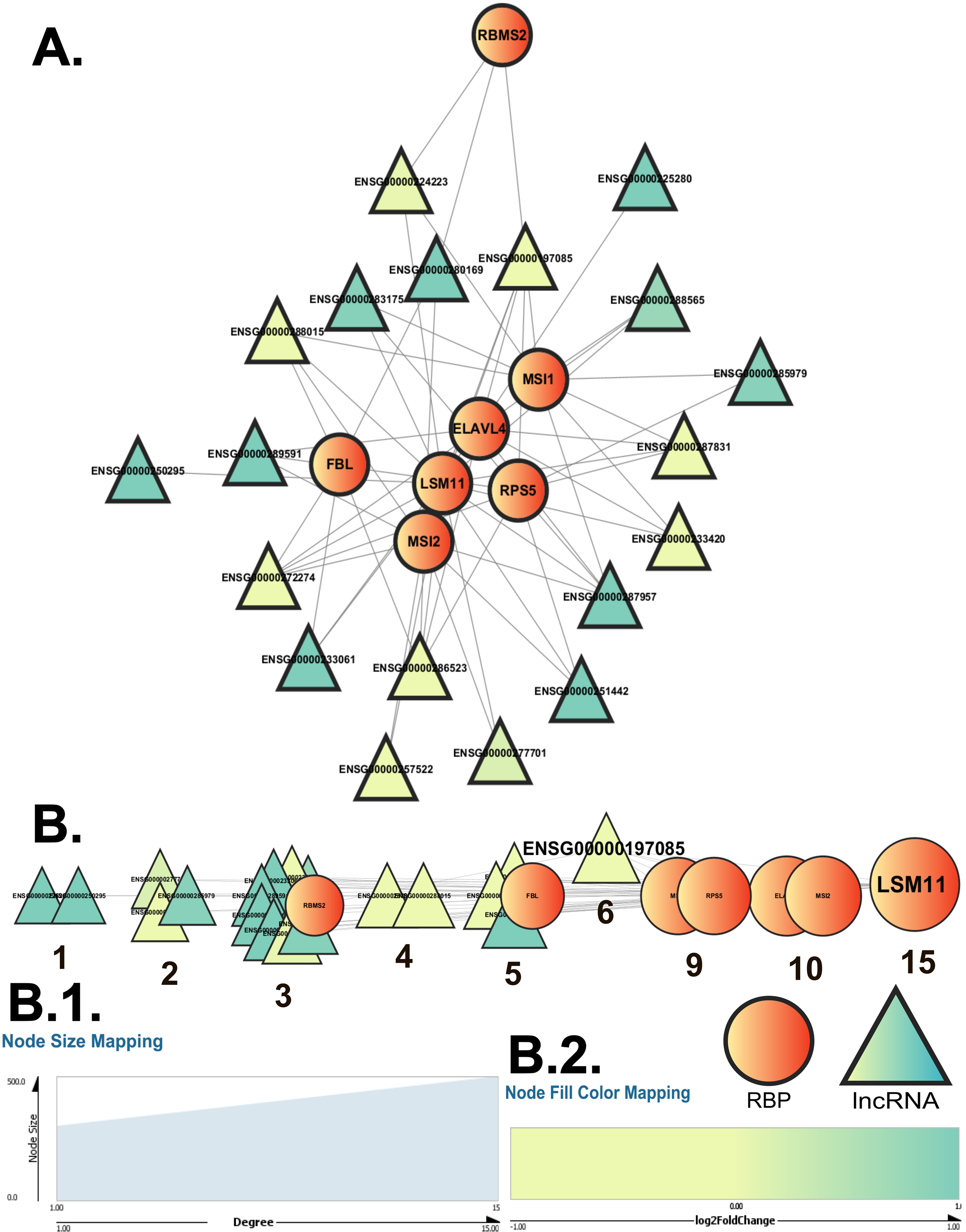

Supplement: Supplementary file 7 — (PNG 1193 kb) [file 12031_2024_2244_Fig17_ESM.png]

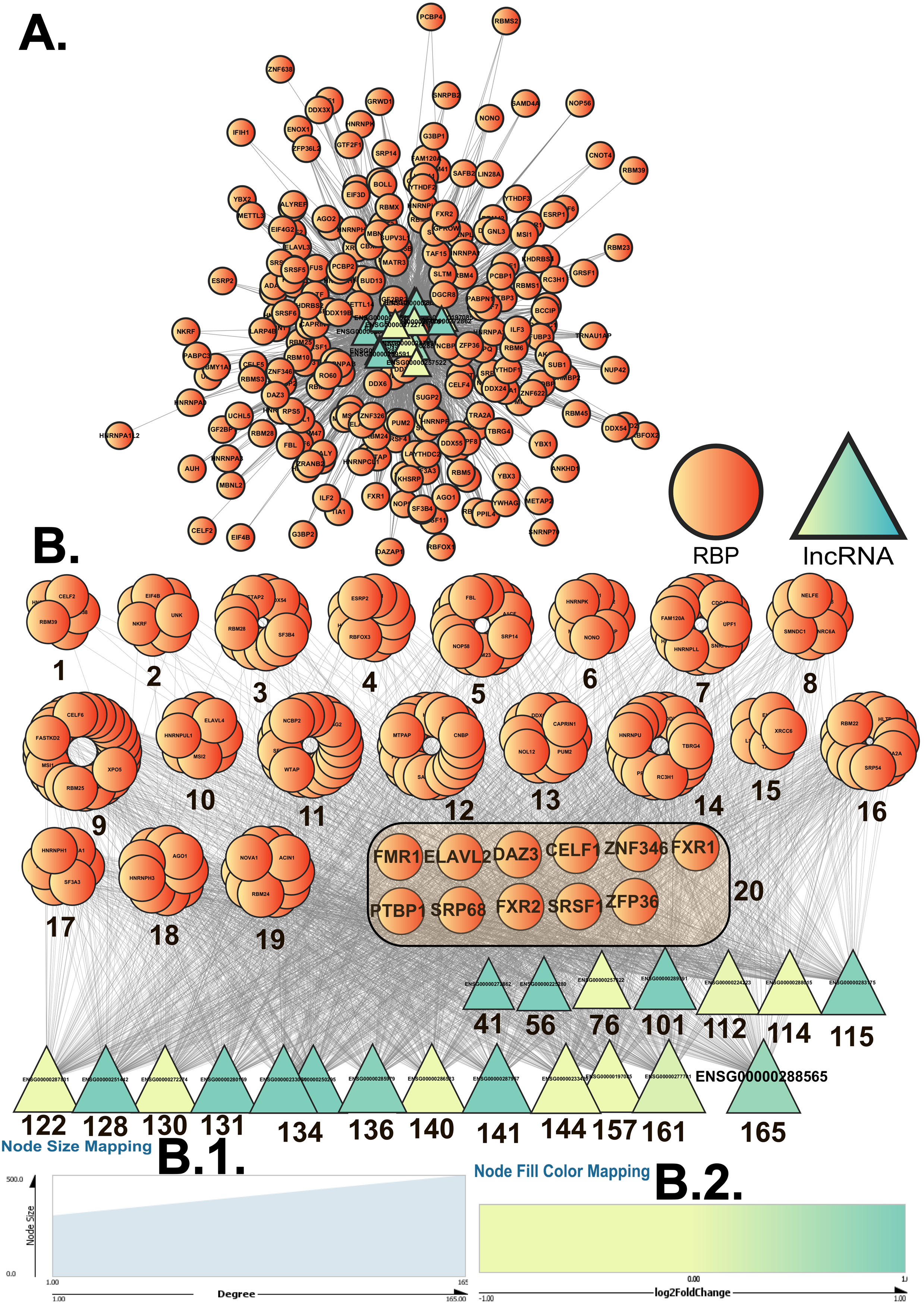

Supplement: Supplementary file 9 — (PNG 4607 kb) [file 12031_2024_2244_Fig18_ESM.png]

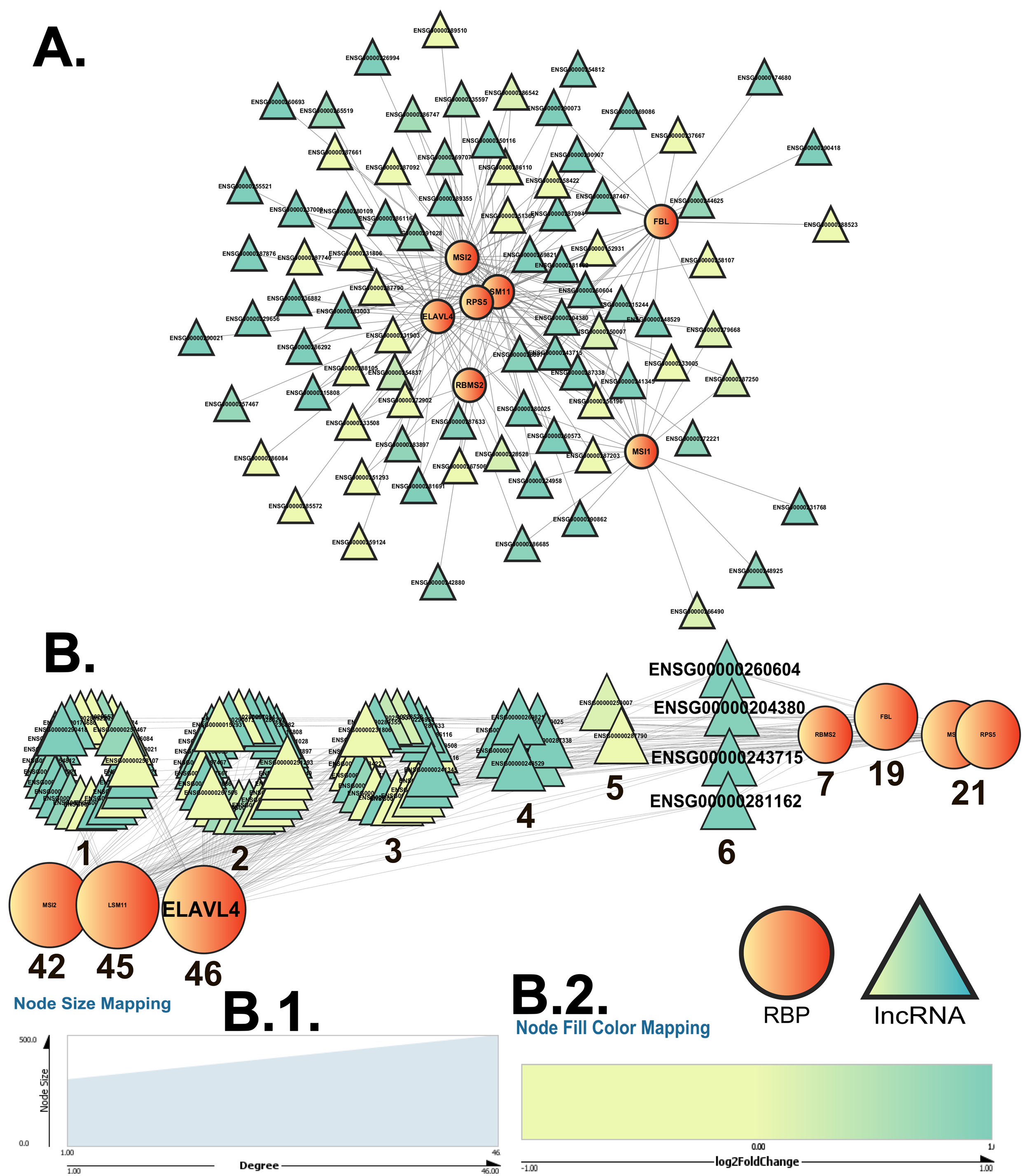

Supplement: Supplementary file 11 — (PNG 1808 kb) [file 12031_2024_2244_Fig19_ESM.png]

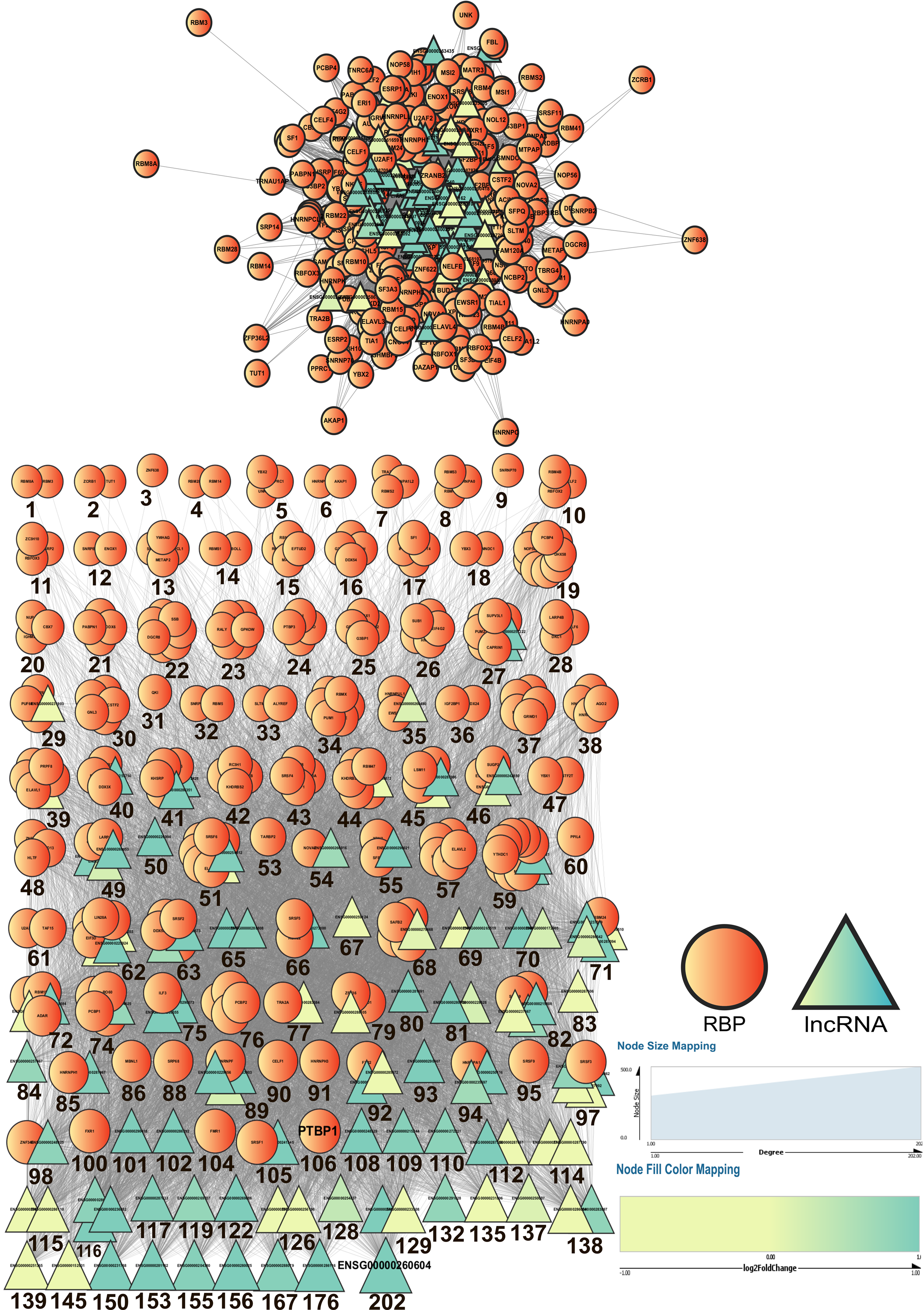

Supplement: Supplementary file 13 — (PNG 4469 kb) [file 12031_2024_2244_Fig20_ESM.png]

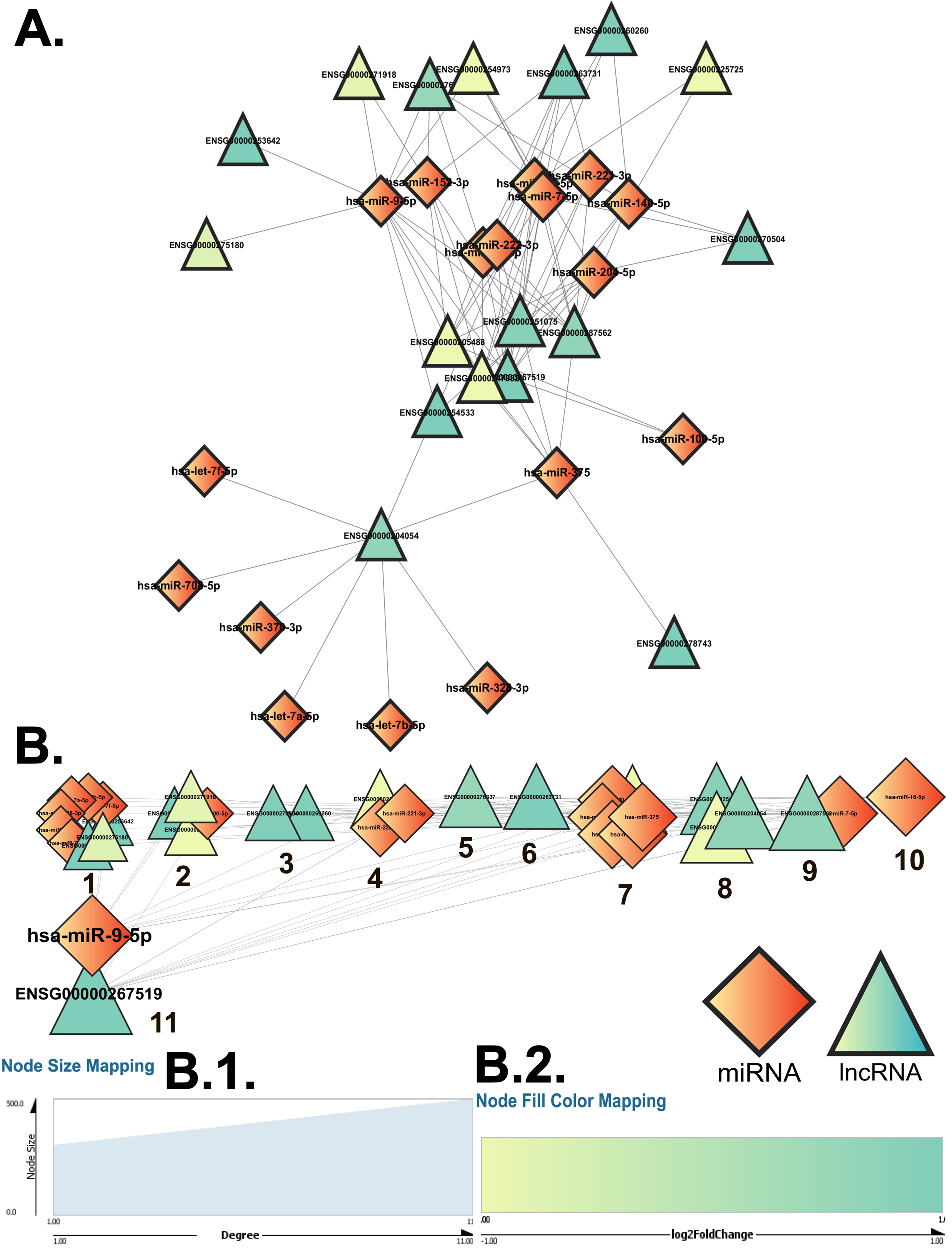

Supplement: Supplementary file 15 — (PNG 1257 kb) [file 12031_2024_2244_Fig21_ESM.png]

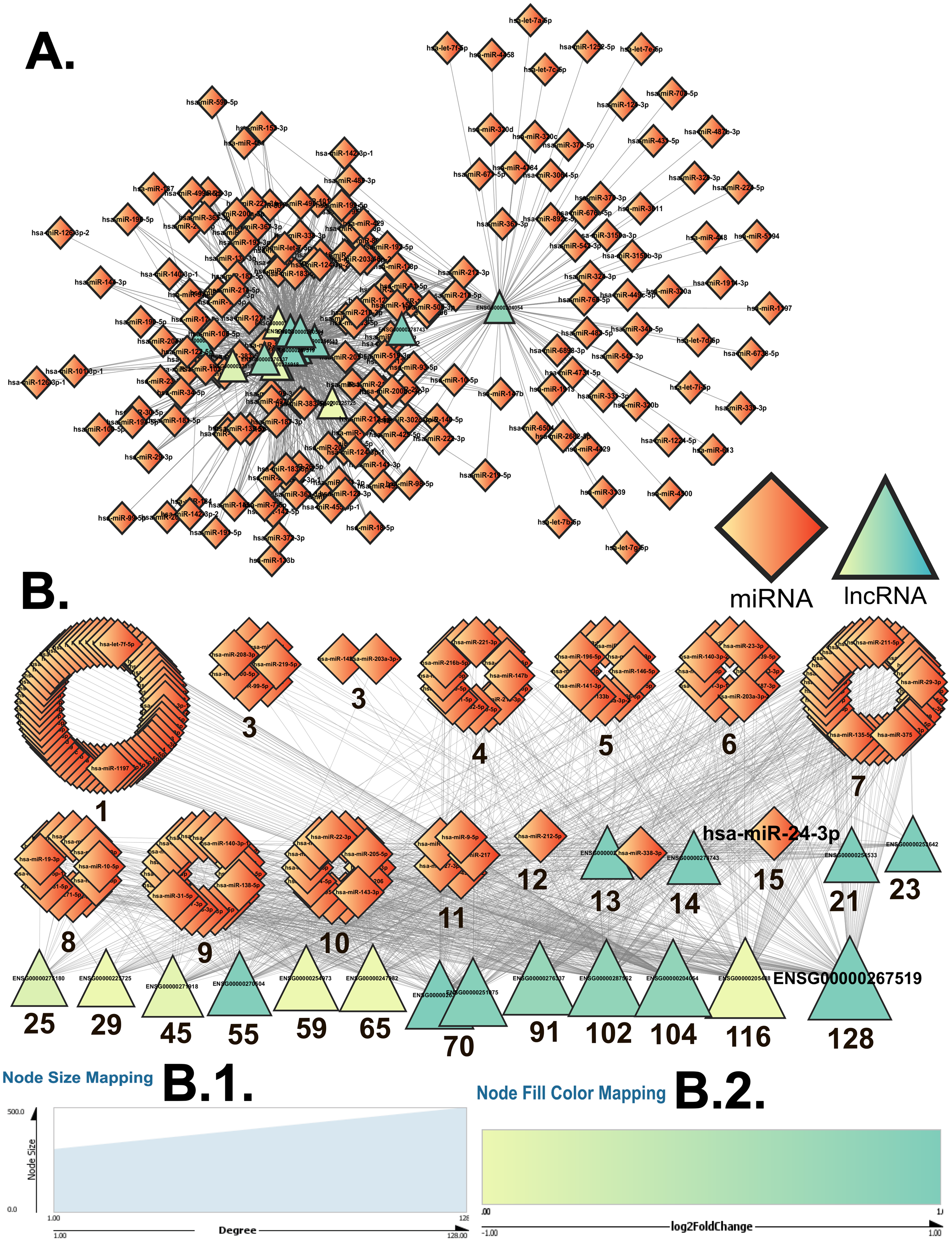

Supplement: Supplementary file 17 — (PNG 4076 kb) [file 12031_2024_2244_Fig22_ESM.png]

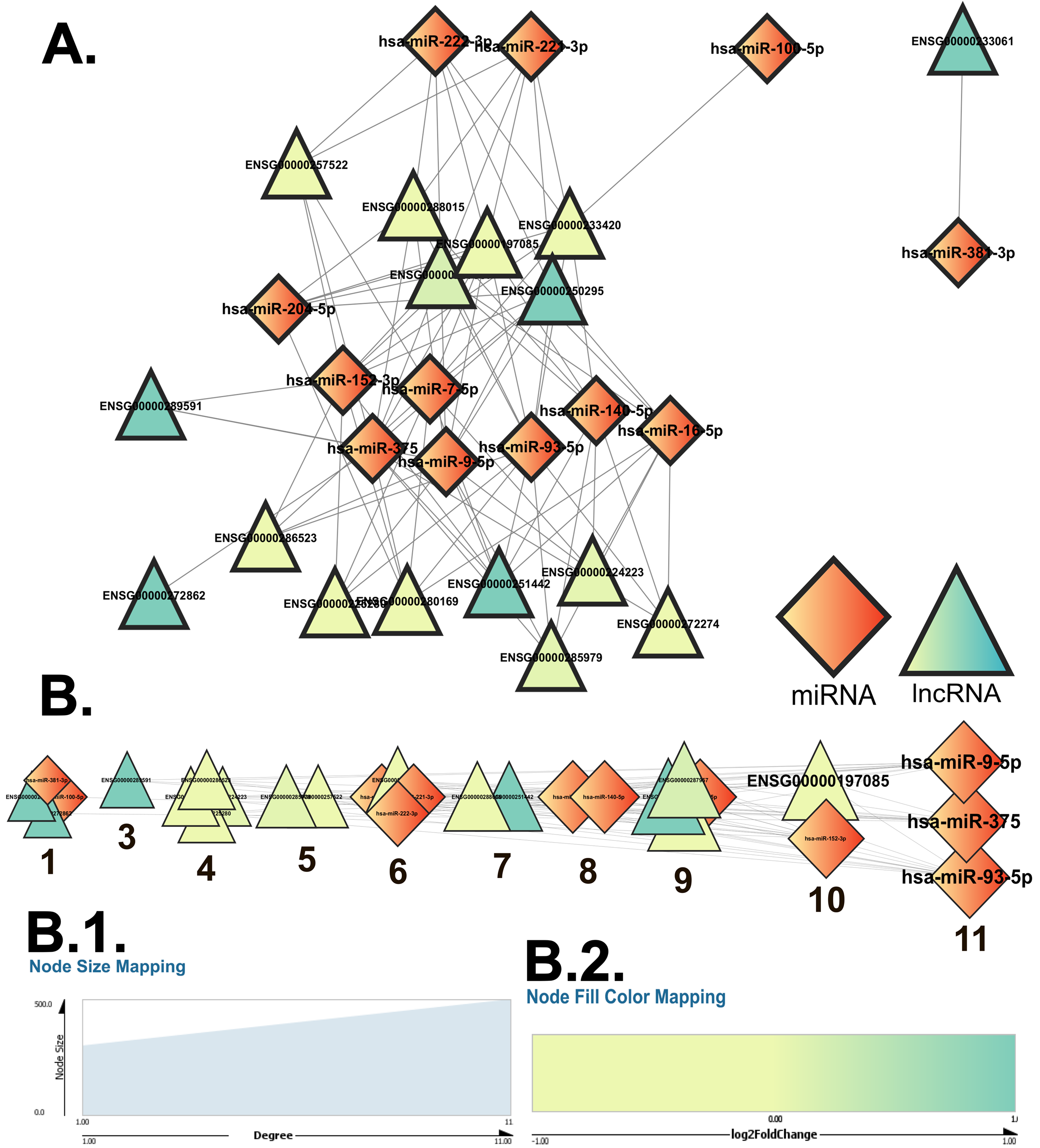

Supplement: Supplementary file 19 — (PNG 1220 kb) [file 12031_2024_2244_Fig23_ESM.png]

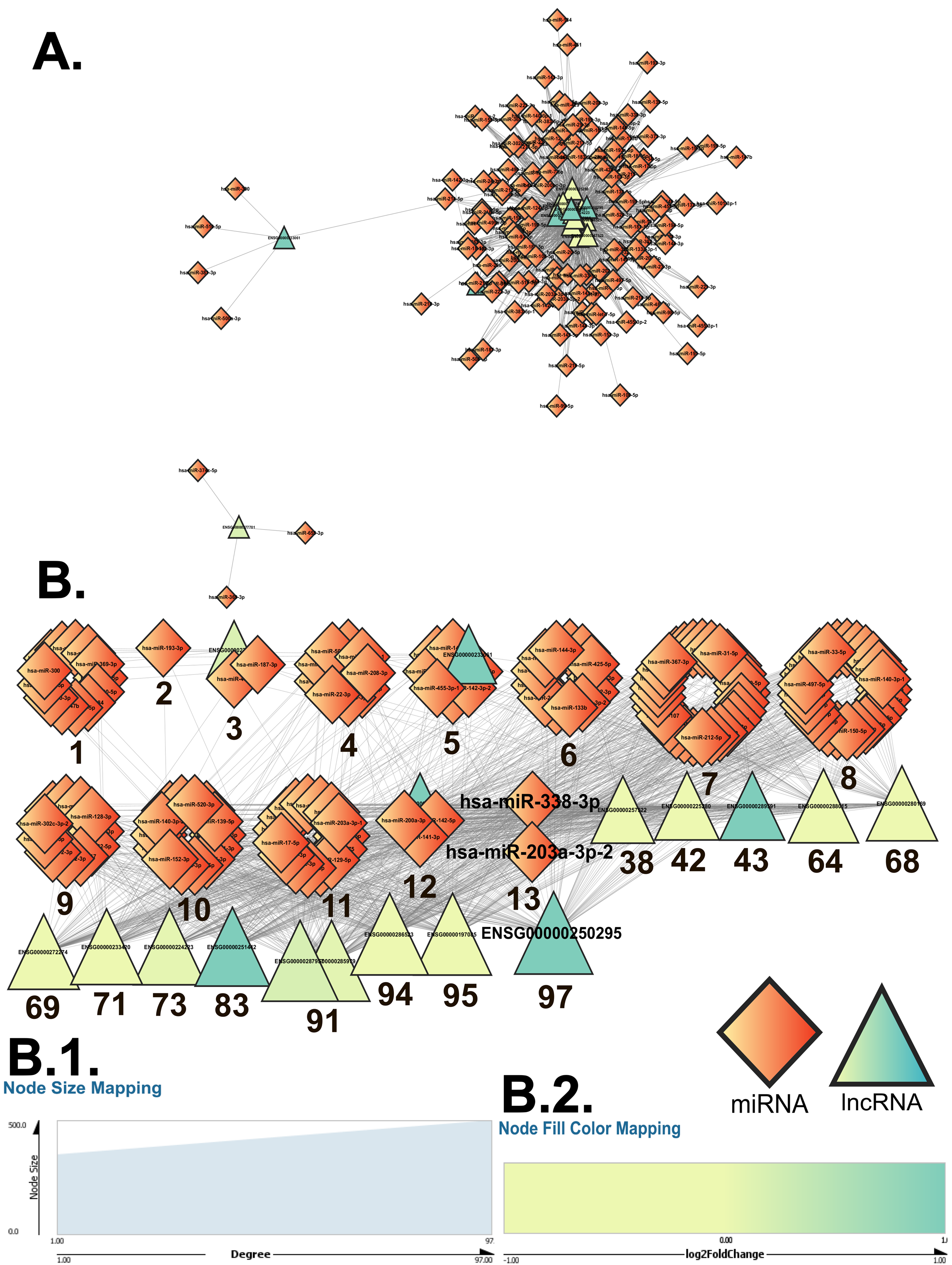

Supplement: Supplementary file 21 — (PNG 2643 kb) [file 12031_2024_2244_Fig24_ESM.png]

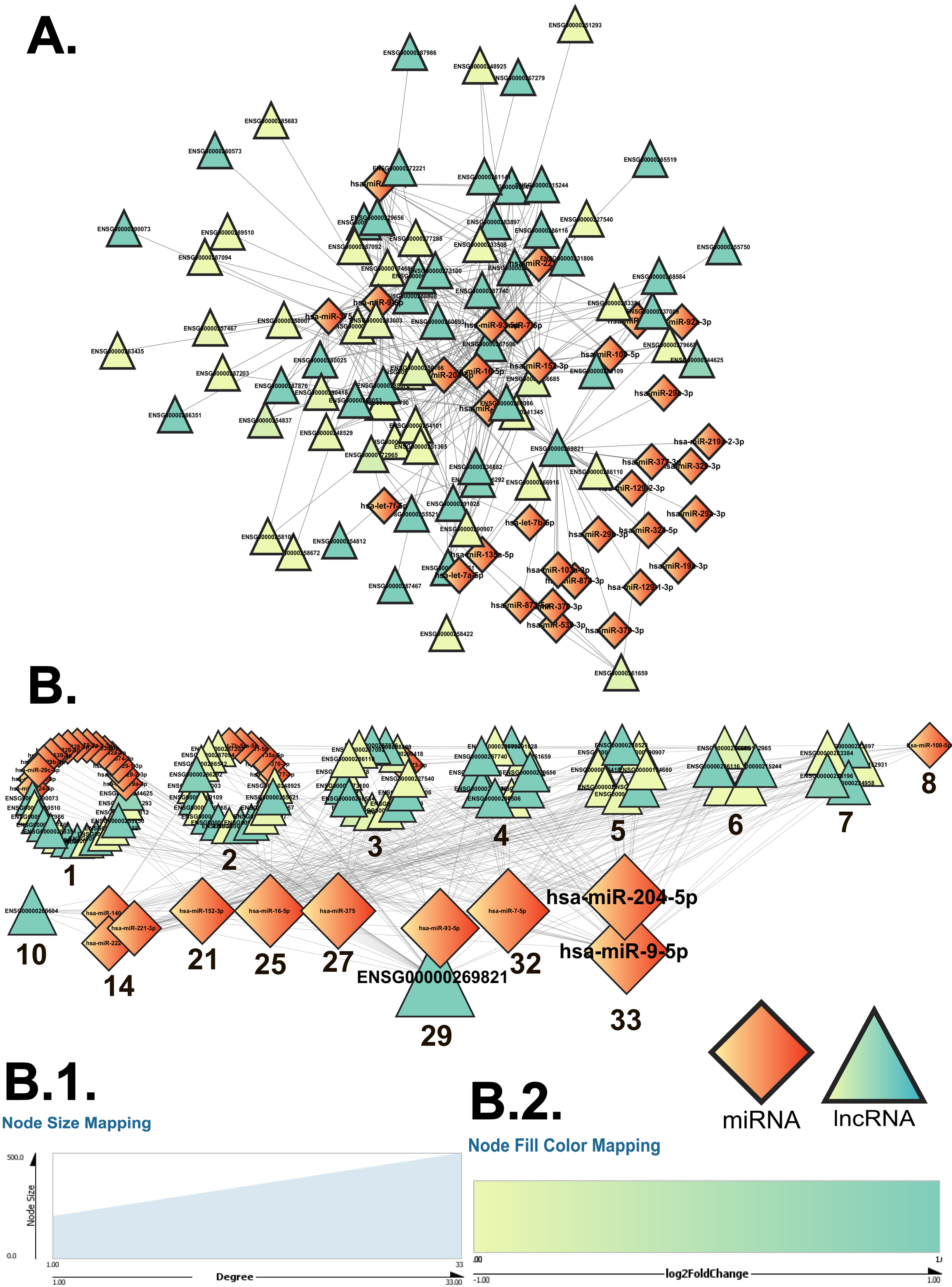

Supplement: Supplementary file 23 — (PNG 2316 kb) [file 12031_2024_2244_Fig25_ESM.png]

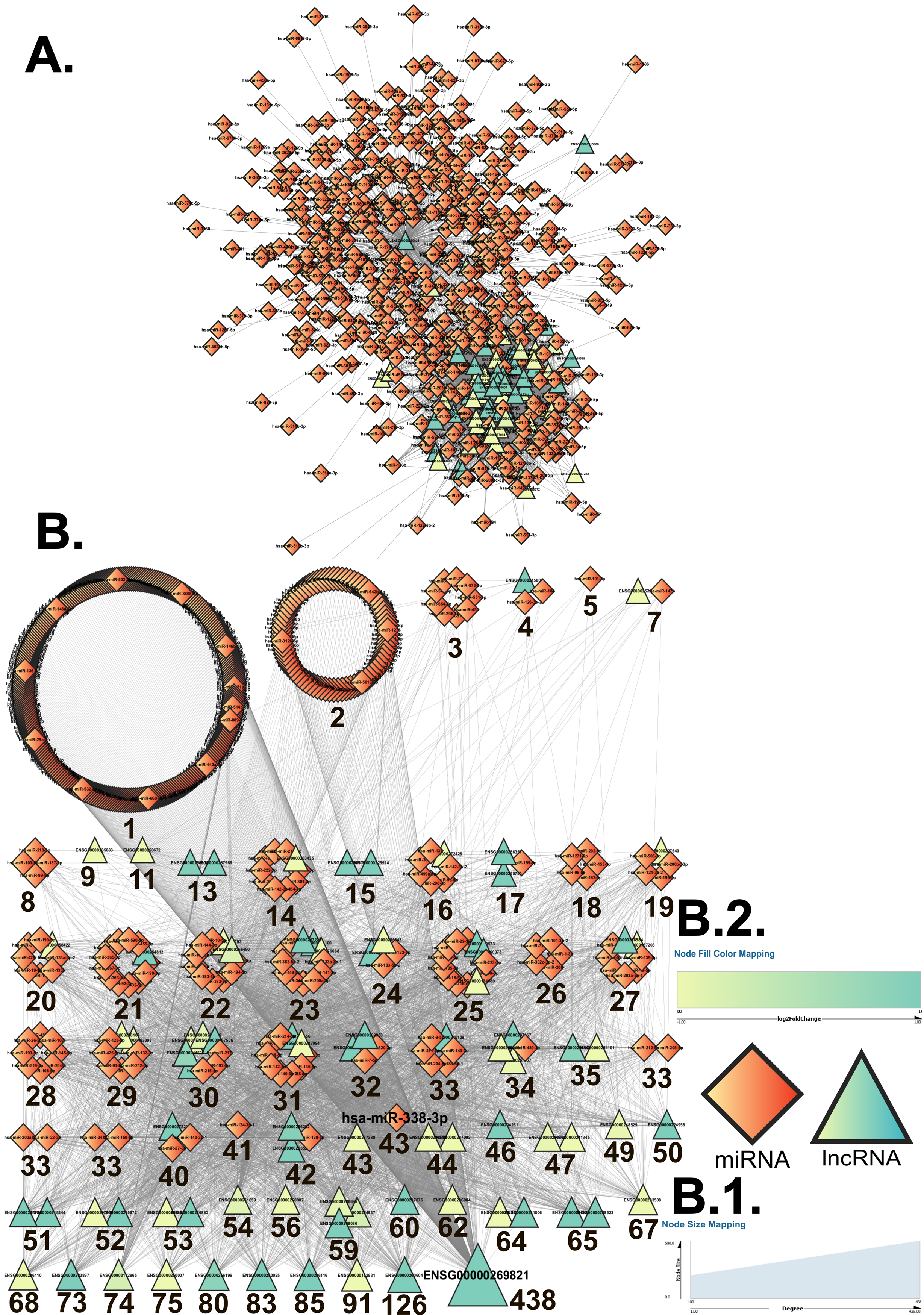

Supplement: Supplementary file 25 — (PNG 5486 kb) [file 12031_2024_2244_Fig26_ESM.png]

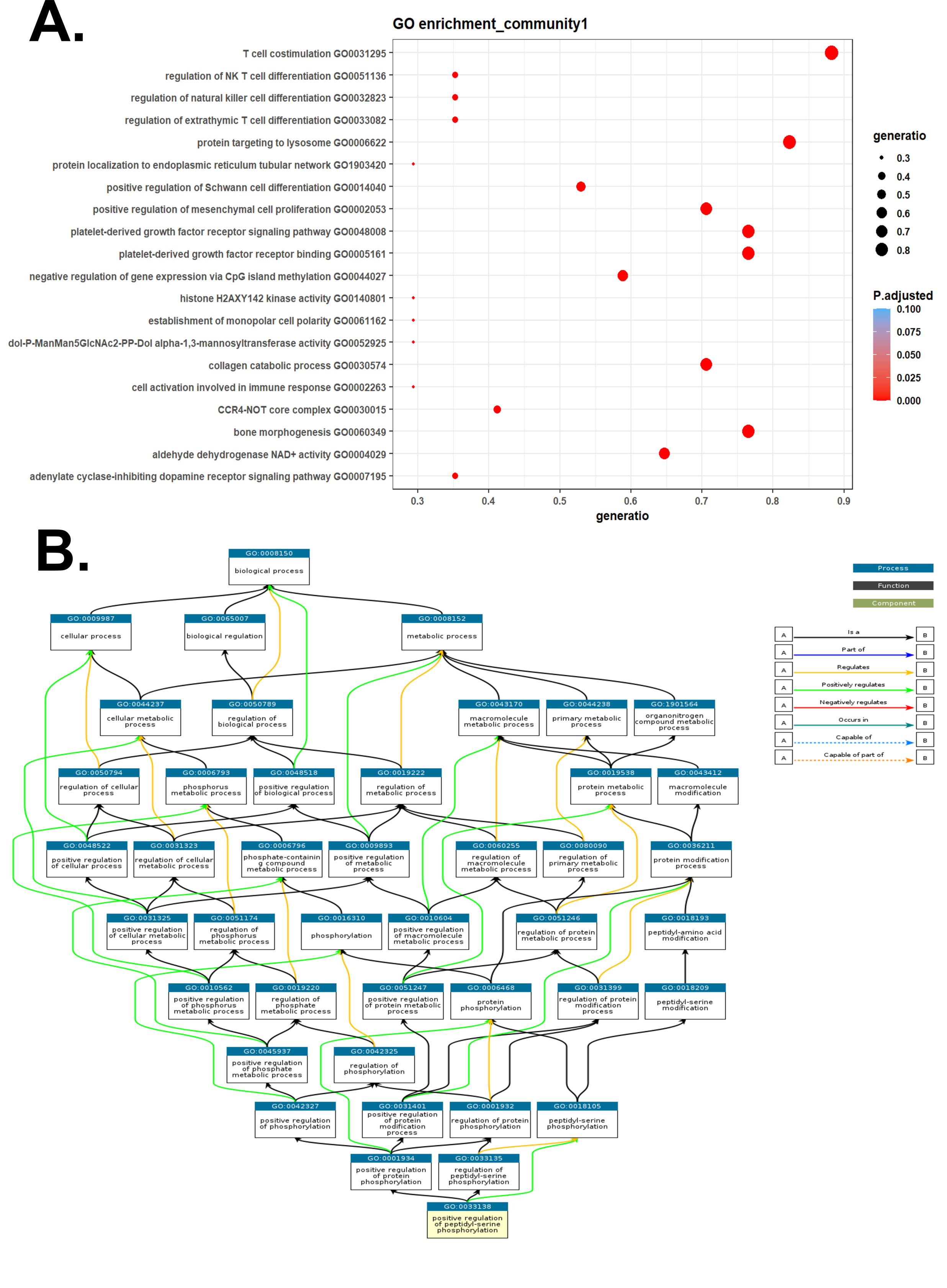

Supplement: Supplementary file 27 — (PNG 1496 kb) [file 12031_2024_2244_Fig27_ESM.png]

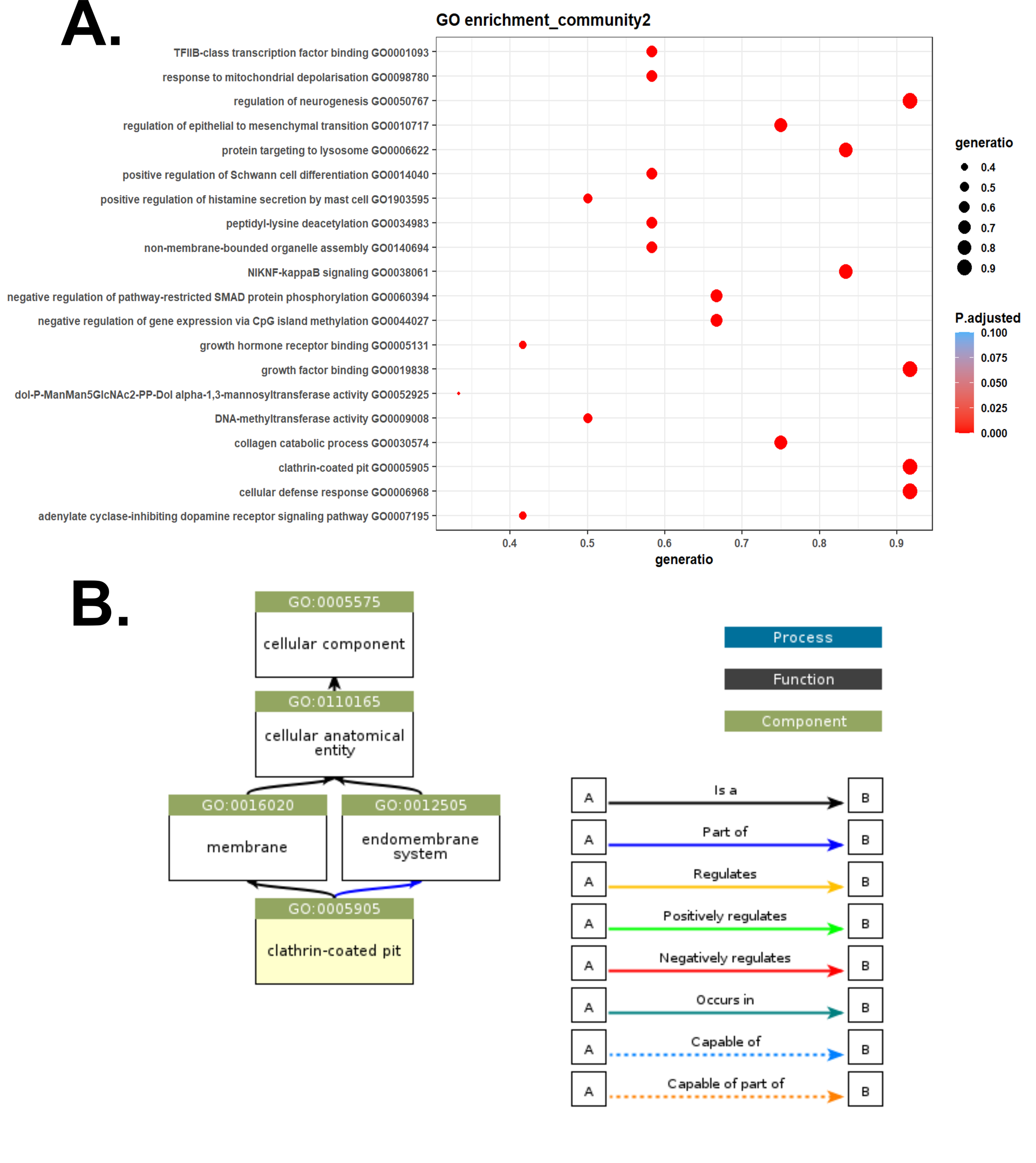

Supplement: Supplementary file 29 — (PNG 852 kb) [file 12031_2024_2244_Fig28_ESM.png]

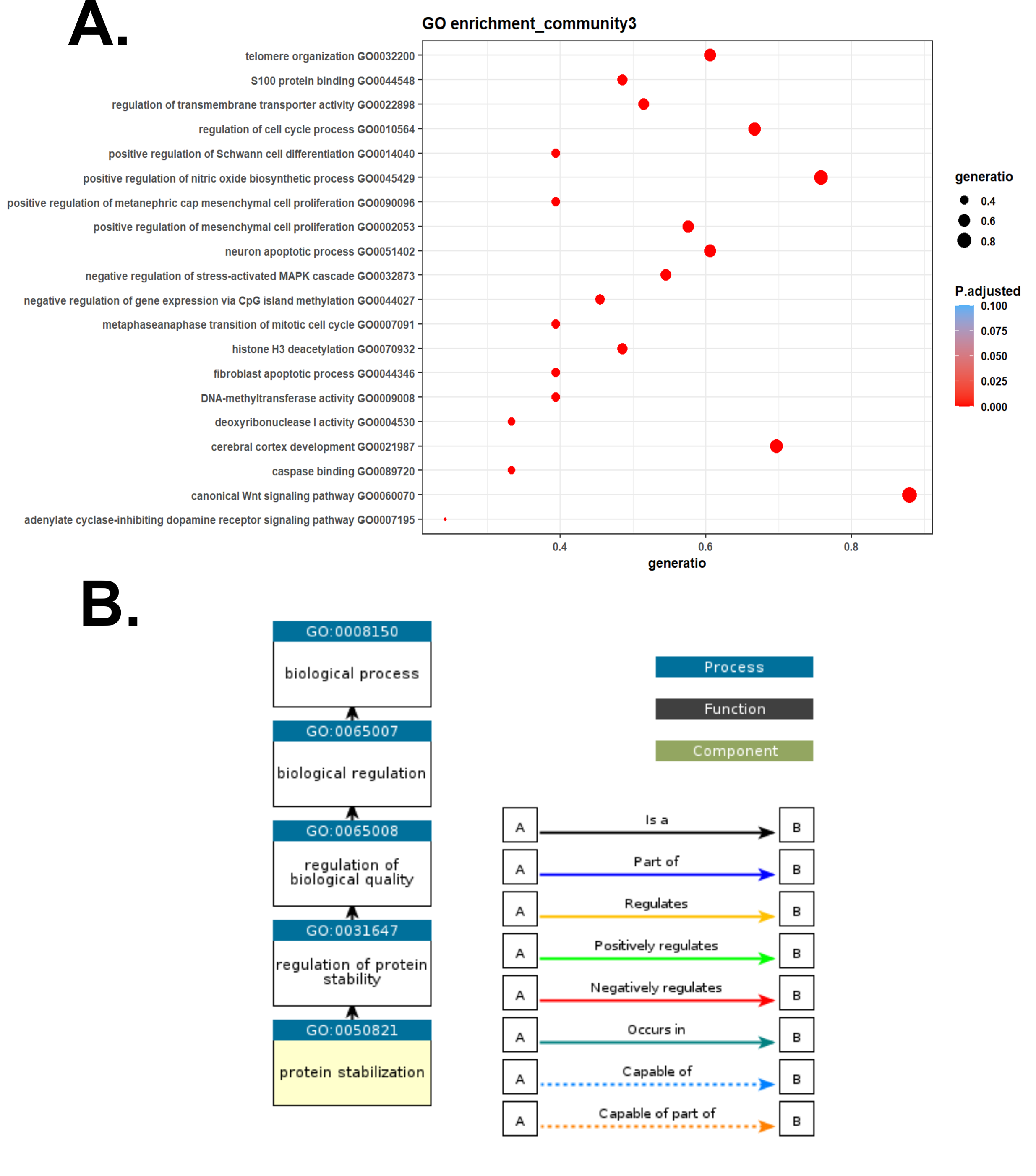

Supplement: Supplementary file 31 — (PNG 839 kb) [file 12031_2024_2244_Fig29_ESM.png]
