# Supplementary material for: A Holistic Analysis of Alzheimer’s Disease-Associated lncRNA Communities Reveals Enhanced lncRNA-miRNA-RBP Regulatory Triad Formation Within Functionally Segregated Clusters
Source: J Mol Neurosci. 2024 Aug 15;74(3):77. doi: 10.1007/s12031-024-02244-0 (PMC11324768; doi:10.1007/s12031-024-02244-0)
Supplement: Supplementary file 38 — (DOCX 12 kb) [file 12031_2024_2244_MOESM22_ESM.docx]

Supplementary Data 1.

#read miR_lnc_RBP data

miR_lnc <- read.csv("[Insert filename containing miR-lnc interaction data]", header = TRUE)

RBP_lnc <- read.csv("[Insert filename containing RBP-lnc interaction data]", header = TRUE)

RBP_miR <- read.csv("[Insert filename containing RBP-miR interaction data]", header = TRUE)

#merge miR_lnc_RBP data

merged_1 <- miR_lnc %>% right_join(RBP_lnc, by = c("[Insert Shared Colname]"))

merged_2 <- merged_1 %>% right_join(RBP_miR, by = c("[Insert Shared Colname]"))

write.csv(merged_2, file = "miR_lnc_RBP_miR.csv")

#match miRNAs

df <- read.csv("miR_lnc_RBP_miR.csv", header = TRUE)

df <- as.data.frame(df)

# Function to compare values and create 'match' column. The match column shows the triads of miR_lnc_RBPs having inter-regulatory relationship.

compare_and_create_match_column <- function(data) {

data$match <- ifelse(data$miRNA.x == data$miRNA.y, "yes", "no")

return(data)

}

# Applying the function to the data frame

df <- compare_and_create_match_column(df)

# Displaying the result

print(df)

# Extracting rows with 'match' column equal to 'yes'

matching_rows <- df[df$match == "yes", ]

# Displaying the matching rows

print("Matching Rows:")

print(matching_rows)

# Assembling a new data frame with columns from matching rows

new_df <- data.frame(matching_rows)

# Displaying the new data frame having only the mathcing rows showing all the miR_lnc_RBP triads

print("New Data Frame:")

print(new_df)
