# Supplementary material for: A Holistic Analysis of Alzheimer’s Disease-Associated lncRNA Communities Reveals Enhanced lncRNA-miRNA-RBP Regulatory Triad Formation Within Functionally Segregated Clusters
Source: J Mol Neurosci. 2024 Aug 15;74(3):77. doi: 10.1007/s12031-024-02244-0 (PMC11324768; doi:10.1007/s12031-024-02244-0)
Supplement: Supplementary file 39 — (DOCX 19 kb) [file 12031_2024_2244_MOESM23_ESM.docx]

====== LSM11/ENSG00000263571 docking ======

# LSM11 interface residue(s):

| PRO | 33A | 2.736 |
| --- | --- | --- |
| LEU | 34A | 4.511 |
| ARG | 174A | 2.372 |
| THR | 175A | 3.067 |
| PHE | 176A | 3.911 |
| ARG | 180A | 1.519 |
| GLU | 203A | 4.421 |
| TYR | 205A | 3.934 |
| LYS | 207A | 4.103 |
| PRO | 208A | 2.703 |
| LEU | 210A | 2.947 |
| LYS | 212A | 3.475 |
| ALA | 213A | 4.177 |
| ARG | 216A | 3.740 |
| ASP | 247A | 4.964 |
| GLN | 255A | 4.223 |
| TRP | 259A | 2.764 |
| LYS | 330A | 1.376 |
| TYR | 335A | 3.815 |
| ARG | 341A | 2.283 |
| ILE | 343A | 4.448 |
| GLN | 345A | 3.630 |
| ILE | 346A | 3.473 |
| PHE | 347A | 2.639 |
| ARG | 349A | 3.460 |
| ASN | 352A | 3.334 |

# ENSG00000263571 interface residue(s):

C 251A 3.740

C 252A 3.475

A 253A 3.239

G 254A 1.376

C 255A 4.157

C 1044A 4.263

U 1045A 2.736

A 1049A 4.448

C 1050A 2.639

C 1051A 3.051

U 1052A 3.643

U 1059A 3.788

C 1060A 3.227

C 1376A 4.964

A 1613A 4.353

C 1614A 2.947

G 1615A 2.372

G 1616A 2.097

G 1617A 1.519

| G | 1618A | 2.283 |
| --- | --- | --- |
| U | 2444A | 4.223 |
| G | 2464A | 2.764 |
| G | 2465A | 3.564 |

#LSM11/ENSG00000263571 interface residue pair(s):

|  |  |  |  |
| --- | --- | --- | --- |
| 33A - 1044A | 4.263 |  |  |
| 33A - 1045A | 2.736 |  |  |
| 34A - 1045A | 4.511 |  |  |
| 174A - 1615A | 2.372 |  |  |
| 175A - 1615A | 3.067 |  |  |
| 176A - 1615A | 3.911 |  |  |
| 180A - 1615A | 4.774 |  |  |
| 180A - 1616A | 2.097 |  |  |
| 180A - 1617A | 1.519 |  |  |
| 203A - 1617A | 4.421 |  |  |
| 205A - 1617A | 3.934 |  |  |
| 205A - 1618A | 4.542 |  |  |
| 207A - 1616A | 4.103 |  |  |
| 207A - 1617A | 4.611 |  |  |
| 208A - 1614A | 4.095 |  |  |
| 208A - 1615A | 3.383 |  |  |
| 208A - 1616A | 2.703 |  |  |
| 210A - 1059A | 3.788 |  |  |
| 210A - 1613A | 4.353 |  |  |
| 210A - 1614A | 2.947 |  |  |
| 212A - 252A | 3.475 |  |  |
| 213A - 252A | 4.177 |  |  |
| 216A - 251A | 3.740 |  |  |
| 216A - 252A | 4.192 |  |  |
| 247A - 1376A | 4.964 |  |  |
| 255A - 2444A | 4.223 |  |  |
| 259A - 2464A | 2.764 |  |  |
| 259A - 2465A | 3.564 |  |  |
| 330A - 253A | 3.239 |  |  |
| 330A - 254A | 1.376 |  |  |
| 330A - 255A | 4.157 |  |  |
| 330A - 1059A | 4.941 |  |  |
| 330A - 1060A | 3.227 |  |  |
| 335A - 1059A | 3.815 |  |  |
| 341A - 1050A | 4.889 |  |  |
| 341A - 1617A | 3.399 |  |  |
| 341A - 1618A | 2.283 |  |  |
| 343A - 1049A | 4.448 |  |  |
| 343A - 1050A | 4.943 |  |  |
| 345A - 1049A | 4.616 |  |  |
| 345A - 1050A | 3.630 |  |  |
| 346A - 1050A | 3.473 |  |  |
| 347A - 1050A | 2.639 |  |  |
| 347A - 1051A | 3.051 |  |  |

| 349A | - 1051A | 3.460 |
| --- | --- | --- |
| 349A | - 1052A | 3.643 |
| 352A | - 1615A | 3.334 |
